# Publisher Correction: Orthogonal Cas9-Cas9 chimeras provide a versatile platform for genome editing

**DOI:** 10.1038/s41467-018-07776-9

**Published:** 2018-12-10

**Authors:** Mehmet Fatih Bolukbasi, Pengpeng Liu, Kevin Luk, Samantha F. Kwok, Ankit Gupta, Nadia Amrani, Erik J. Sontheimer, Lihua Julie Zhu, Scot A. Wolfe

**Affiliations:** 1 Department of Molecular, Cell and Cancer Biology, University of Massachusetts Medical School, Worcester, MA USA; 2 Department of Biochemistry and Molecular Pharmacology, University of Massachusetts Medical School, Worcester, MA USA; 3 RNA Therapeutics Institute, University of Massachusetts Medical School, Worcester, MA USA; 4 Program in Molecular Medicine, University of Massachusetts Medical School, Worcester, MA USA; 5 Program in Bioinformatics and Integrative Biology, University of Massachusetts Medical School, Worcester, MA USA; 6 Present Address: Exonics Therapeutics, Watertown, MA USA; 7 Present Address: Bluebird Bio., Cambridge, MA USA

Correction to: *Nature Communications*; 10.1038/s41467-018-07310-x; published online 19 November 2018

The original version of this Article contained errors in the author affiliations. Mehmet Fatih Bolukbasi was incorrectly associated with Bluebird Bio., Cambridge, MA, USA and Ankit Gupta was incorrectly associated with Exonics Therapeutics, Watertown, MA, USA. This has now been corrected in the HTML version of the Article. The PDF version of the Article was correct at the time of publication.

