# Supplementary material for: Orthogonal Cas9–Cas9 chimeras provide a versatile platform for genome editing
Source: Nat Commun. 2018 Nov 19;9:4856. doi: 10.1038/s41467-018-07310-x (PMC6242970; doi:10.1038/s41467-018-07310-x)
Supplement: Supplementary file 1 — Supplementary Information [file 41467_2018_7310_MOESM1_ESM.pdf]

Supplementary Information

Orthogonal Cas9-Cas9 chimeras provide a versatile platform for genome editing

Bolukbasi *et al*.

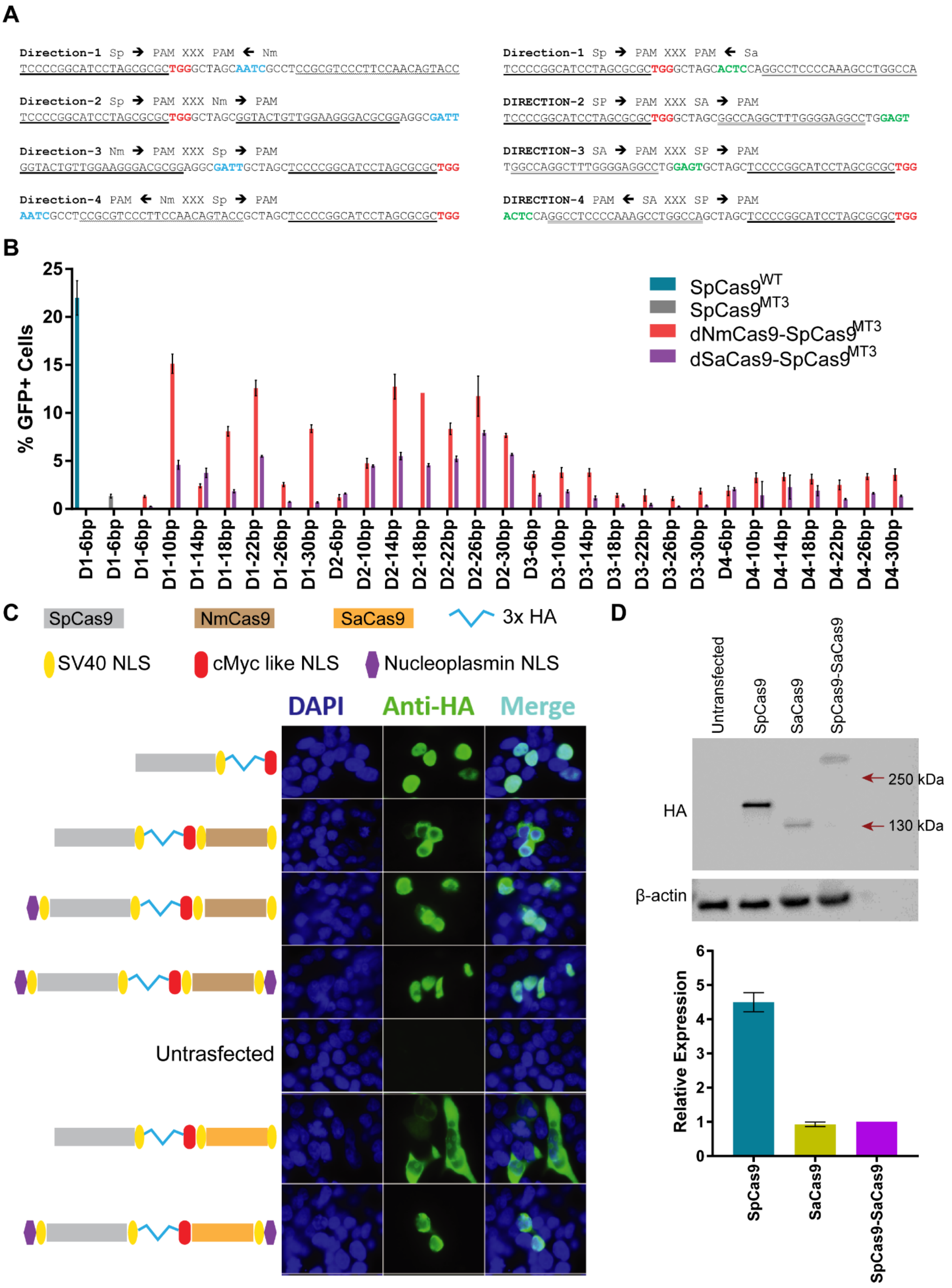
A
Direction-1 Sp → PAM XXX PAM ← Nm
TCCCCGGCATCCTAGCGCGCTGGGCTAGCAATCGCCTCCGCGTCCCTTCCAACAGTACC
Direction-2 Sp → PAM XXX Nm → PAM
TCCCCGGCATCCTAGCGCGCTGGGCTAGCGGTACTGTTGGAAGGGACGCGGAGGCGATT
Direction-3 Nm → PAM XXX Sp → PAM
GGTACTGTTGGAAGGGACGCGGAGGCGATTGCTAGCTCCCCGGCATCCTAGCGCGCTGG
Direction-4 PAM ← Nm XXX Sp → PAM
AATCGCCTCCGCGTCCCTTCCAACAGTACCGCTAGCTCCCCGGCATCCTAGCGCGCTGG
Direction-1 Sp → PAM XXX PAM ← Sa
TCCCCGGCATCCTAGCGCGCTGGGCTAGCACTCCAGGCCTCCCCAAAGCCTGGCCA
DIRECTION-2 SP → PAM XXX SA → PAM
TCCCCGGCATCCTAGCGCGCTGGGCTAGCGGCCAGGCTTTGGGGAGGCCTGGAGT
DIRECTION-3 SA → PAM XXX SP → PAM
TGGCCAGGCTTTGGGGAGGCCTGGAGTGCTAGCTCCCCGGCATCCTAGCGCGCTGG
DIRECTION-4 PAM ← SA XXX SP → PAM
ACTCCAGGCCTCCCCAAAGCCTGGCCAGCTAGCTCCCCGGCATCCTAGCGCGCTGG
B
% GFP+ Cells
25
20
15
10
5
0
SpCas9WT
SpCas9MT3
dNmCas9-SpCas9MT3
dSaCas9-SpCas9MT3
D1-6bp
D1-6bp
D1-6bp
D1-10bp
D1-14bp
D1-18bp
D1-22bp
D1-26bp
D1-30bp
D2-6bp
D2-10bp
D2-14bp
D2-18bp
D2-22bp
D2-26bp
D2-30bp
D3-6bp
D3-10bp
D3-14bp
D3-18bp
D3-22bp
D3-26bp
D3-30bp
D4-6bp
D4-10bp
D4-14bp
D4-18bp
D4-22bp
D4-26bp
D4-30bp
C
SpCas9
NmCas9
SaCas9
3x HA
SV40 NLS
cMyc like NLS
Nucleoplasmin NLS
DAPI
Anti-HA
Merge
Untrasfected
D
Untransfected
SpCas9
SaCas9
SpCas9-SaCas9
HA
250 kDa
130 kDa
β-actin
Relative Expression
6
5
4
3
2
1
0
SpCas9
SaCas9
SpCas9-SaCas9

Supplementary Figure 1 | Identification of the functional parameters for Cas9-Ca9 fusion activity. (**a**) Sequence information of the target sites tested for SpCas9-SaCas9, SaCas9-SpCas9, SpCas9-NmCas9 and NmCas9-SpCas9 fusions. SpCas9 protospacer is bold underlined and its PAM is in red. SaCas9 protospacer is double underlined and its PAM is green. NmCas9 protospacer is wavy underlined and its PAM is in blue. (**b**) Activity profiles of SpCas9 (blue), SpCas9^{MT3} (R1335K; gray), and N-terminal fusions for dNmCas9-SpCas9^{MT3} (pink), and dSaCas9-SpCas9^{MT3} (purple) in the GFP reporter assay. The data are from three independent biological replicates performed on different days in HEK293T cells. Error bars indicate ±s.e.m. (**c**) Subcellular localization of Cas9-Cas9 fusion proteins. On the left is the schematic of various compositions of the Cas9-Cas9 fusion protein. On the right, immunofluorescence imaging of nucleus (blue) and Cas9-Cas9 proteins (green). The Cas9-Cas9 fusions primarily localize to cytoplasm when the NLSes are only in the linker (top second and top sixth panel), whereas to they localize to the nucleus with the addition of terminal NLSes. (**d**) Expression of various Cas9 nucleases. Top, a representative western blot gel image for HA-tag (Cas9 epitope tag) and β-actin that indicate nuclease expression and loading control respectively. Arrowheads point the corresponding molecular size based on molecular ladder used (not shown). Bottom, quantification of the western blot band intensities for each nuclease, where the bar value indicates fold expression relative to SpCas9-SaCas9 expression for each sample normalized to β-actin. The data are from three independent biological replicates performed on different days in HEK293T cells. Error bars indicate ±s.e.m.

A

SaCas9_sgRNA_#1 D1-34bp
GTCCCCTCCACCCCACAGTG**GGG**CCACTAGGGACAGGATTGGTGACAGAAAAGCCCC**ATCC**TTAGGCCTCCTCCTTCCTAGTCTCC

SaCas9_sgRNA_#2 D2-46bp
GTCCCCTCCACCCCACAGTG**GGG**CCACTAGGGACAGGATTGGTGACAGAAAAGCCCCATCCTTAGGCCTCCTCCTTCCTAGTCTCCTGA
TATT**GGGT**

SaCas9_sgRNA_#3 D1-77bp
GTCCCCTCCACCCCACAGTG**GGG**CCACTAGGGACAGGATTGGTGACAGAAAAGCCCCATCCTTAGGCCTCCTCCTTCCTAGTCTCCTGA
TATTGGGTCTA**ACCC**CCACCTCCTGTTAGGCAGATTCC

SaCas9_sgRNA_#4 D1-99bp
GTCCCCTCCACCCCACAGTG**GGG**CCACTAGGGACAGGATTGGTGACAGAAAAGCCCCATCCTTAGGCCTCCTCCTTCCTAGTCTCCTGA
TATTGGGTCTAACCCCCACCTCCTGTTAGGCAG**ATTC**CTTATCTGGTGACACACCCCCATTTC

SaCas9_sgRNA_#5 D1-118bp
GTCCCCTCCACCCCACAGTG**GGG**CCACTAGGGACAGGATTGGTGACAGAAAAGCCCCATCCTTAGGCCTCCTCCTTCCTAGTCTCCTGA
TATTGGGTCTAACCCCCACCTCCTGTTAGGCAGATTCCTTATCTGGTGACAC**ACCC**CCATTTCCTGGAGCCATCTCTCTCCTTGCCAGA
ACCTCTA

SaCas9_sgRNA_#6 D1-190bp
GTCCCCTCCACCCCACAGTG**GGG**CCACTAGGGACAGGATTGGTGACAGAAAAGCCCCATCCTTAGGCCTCCTCCTTCCTAGTCTCCTGA
TATTGGGTCTAACCCCCACCTCCTGTTAGGCAGATTCCTTATCTGGTGACACACCCCCATTTCCTGGAGCCATCTCTCTCCTTGCCAGA
ACCTCTAAGGTTTGCTTACGATGGAGCCAGAGAGG**ATCC**TGGGAGGGAGAGCTTGGCAGGGGG

SaCas9_sgRNA_#7 D2-189bp
GTCCCCTCCACCCCACAGTG**GGG**CCACTAGGGACAGGATTGGTGACAGAAAAGCCCCATCCTTAGGCCTCCTCCTTCCTAGTCTCCTGA
TATTGGGTCTAACCCCCACCTCCTGTTAGGCAGATTCCTTATCTGGTGACACACCCCCATTTCCTGGAGCCATCTCTCTCCTTGCCAGA
ACCTCTAAGGTTTGCTTACGATGGAGCCAGAGAGGATCCTGGGAGGGAGAGCTTGGCAGG**GGGT**

SaCas9_sgRNA_#8 D2-209bp
GTCCCCTCCACCCCACAGTG**GGG**CCACTAGGGACAGGATTGGTGACAGAAAAGCCCCATCCTTAGGCCTCCTCCTTCCTAGTCTCCTGA
TATTGGGTCTAACCCCCACCTCCTGTTAGGCAGATTCCTTATCTGGTGACACACCCCCATTTCCTGGAGCCATCTCTCTCCTTGCCAGA
ACCTCTAAGGTTTGCTTACGATGGAGCCAGAGAGGATCCTGGGAGGGAGAGCTTGGCAGGGGGTGGGAGGGAAGGGGGG**GGAT**

SaCas9_sgRNA_#9 D1-274bp
GTCCCCTCCACCCCACAGTG**GGG**CCACTAGGGACAGGATTGGTGACAGAAAAGCCCCATCCTTAGGCCTCCTCCTTCCTAGTCTCCTGA
TATTGGGTCTAACCCCCACCTCCTGTTAGGCAGATTCCTTATCTGGTGACACACCCCCATTTCCTGGAGCCATCTCTCTCCTTGCCAGA
ACCTCTAAGGTTTGCTTACGATGGAGCCAGAGAGGATCCTGGGAGGGAGAGCTTGGCAGGGGGTGGGAGGGAAGGGGGGGATGCGTGAC
CTGCCCGGTTCTCAGTGGCCACCCTGCGCT**ACCC**TCTCCCAGAACCTGAGCTGCTCTG

SaCas9_sgRNA_#10 D1-328bp
GTCCCCTCCACCCCACAGTG**GGG**CCACTAGGGACAGGATTGGTGACAGAAAAGCCCCATCCTTAGGCCTCCTCCTTCCTAGTCTCCTGA
TATTGGGTCTAACCCCCACCTCCTGTTAGGCAGATTCCTTATCTGGTGACACACCCCCATTTCCTGGAGCCATCTCTCTCCTTGCCAGA
ACCTCTAAGGTTTGCTTACGATGGAGCCAGAGAGGATCCTGGGAGGGAGAGCTTGGCAGGGGGTGGGAGGGAAGGGGGGGATGCGTGAC
CTGCCCGGTTCTCAGTGGCCACCCTGCGCTACCCTCTCCCAGAACCTGAGCTGCTCTGACGCGGCCGTCTGGTGCGTTTCACTG**ATCC**T
GGTGCTGCAGCTTCCTTACACTT

SaCas9_sgRNA_#11 D2-382
GTCCCCTCCACCCCACAGTG**GGG**CCACTAGGGACAGGATTGGTGACAGAAAAGCCCCATCCTTAGGCCTCCTCCTTCCTAGTCTCCTGA
TATTGGGTCTAACCCCCACCTCCTGTTAGGCAGATTCCTTATCTGGTGACACACCCCCATTTCCTGGAGCCATCTCTCTCCTTGCCAGA
ACCTCTAAGGTTTGCTTACGATGGAGCCAGAGAGGATCCTGGGAGGGAGAGCTTGGCAGGGGGTGGGAGGGAAGGGGGGGATGCGTGAC
CTGCCCGGTTCTCAGTGGCCACCCTGCGCTACCCTCTCCCAGAACCTGAGCTGCTCTGACGCGGCCGTCTGGTGCGTTTCACTGATCCT
GGTGCTGCAGCTTCCTTACACTTCCCAAGAGGAGAAGCAGTTTGGAAAAACAAAATCAGAATAAGTTGGTCCCT**GAGT**

B

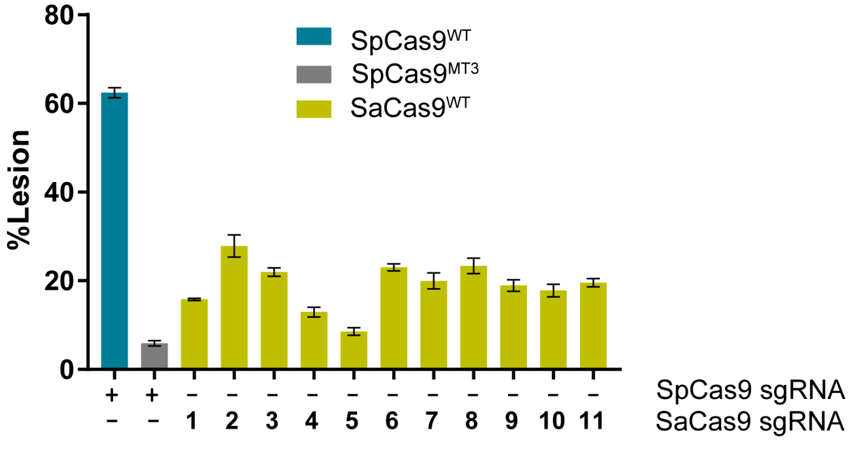

Supplementary Figure 2 | Activity profile analysis of SpCas9-SaCas9 fusions as a function of distance between Cas9 binding sites. (**a**) Sequence information of the *AAVS1* target sites for SpCas9-SaCas9 fusions. The SpCas9 protospacer is bold underlined and its PAM is in red, and the SaCas9 protospacer is double underlined and its PAM is green. (**b**) SaCas9 lesion rates and types are determined by PCR-amplicon deep sequencing. The data are from three independent biological replicates performed on different days in HEK293T cells (**Supplementary Data 1**). Error bars indicate ±s.e.m.

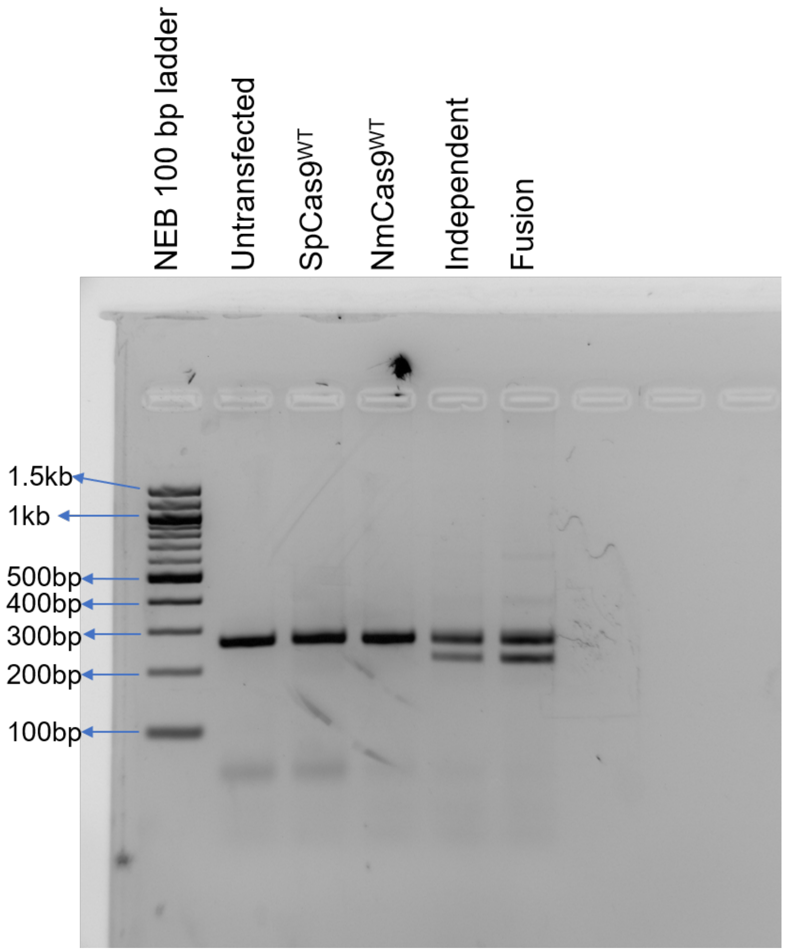

Supplementary Figure 3 | Uncropped gel image from **Fig. 3a**. Genomic region containing the target site is PCR amplified; the higher band is the wild-type sequence or sequences with small indels, and the lower band is the segmental deletion product generated by dual nucleases.

A

VEGFA_TS2

SpCas9_SaCas9-1: GAAAAGTTTCAGTGCGACGCCGCGAGCCCCGACCCCCTCCACCCCGCCTCCGG
SpCas9_SaCas9-2: GACCCCCTCCACCCCGCCTCCGGGCGCGGGCTCCGGCCCCTGCCCGCGGCTCGCCGC
SpCas9_SaCas9-3: GACCCCCTCCACCCCGCCTCCGGGCGCGGGCTCCGGCCCCTGCCCGCGGCTCGCCGCGTCCACTGTCCGCCGC
SpCas9_SaCas9-4: GACCCCCTCCACCCCGCCTCCGGGCGCGGGCTCCGGCCCCTGCCCGCGGCTCGCCGCCGCGTCCACTGTCCGCCGCCGGCCGGGGA

B

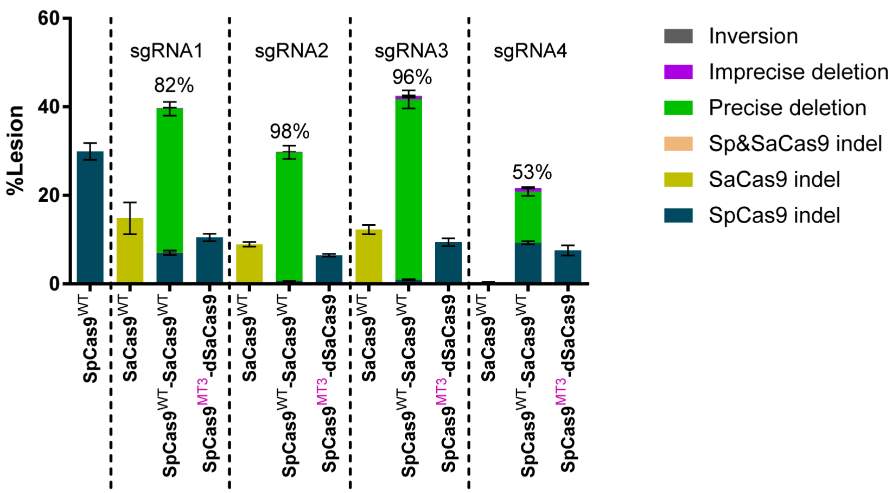

C

VEGFA_TS3

SpCas9_SaCas9-1: GTGAATGGAGCGAGCAGCGTCTTCGAGAGTGAGGACGTGTGTGTCTGTGTGGGTGAGTGAGTGTGTGCGTGTGG
SpCas9_SaCas9-2: GGTGAGTGAGTGTGTGCGTGTGGGGTTGAGGGCGTTGGAGCGGGGAGAAGGCCAGGGGT
SpCas9_SaCas9-3: GGTGAGTGAGTGTGTGCGTGTGGGGTTGAGGGCGTTGGAGCGGGGAGAAGGCCAGGGGTCACTCCAGGAT
SpCas9_SaCas9-4: GGTGAGTGAGTGTGTGCGTGTGGGGTTGAGGGCGTTGGAGCGGGGAGAAGGCCAGGGGTCACTCCAGGATTCCAATAGATCTGTGTGTCC
SpCas9_SaCas9-5: GGTGAGTGAGTGTGTGCGTGTGGGGTTGAGGGCGTTGGAGCGGGGAGAAGGCCAGGGGTCACTCCAGGATTCCAATAGATCTGTGTGTCCCTCTCCCC

D

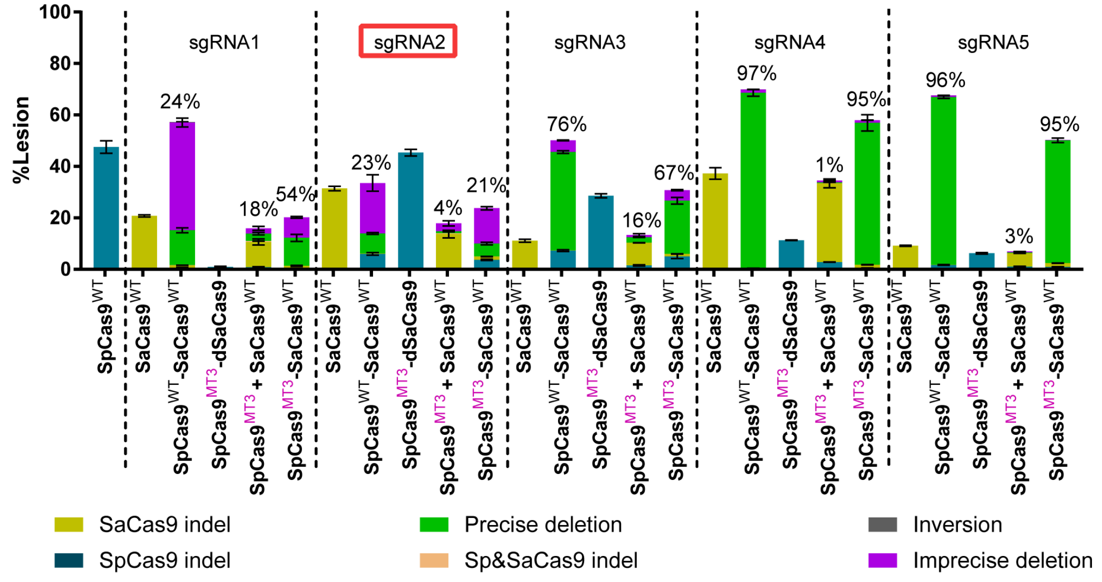

Supplementary Figure 4 | Analysis of lesion type and frequency profile of SpCas9-SaCas9 fusions. (**a**) Sequence information of the *VEGFA-TS2* target sites for SpCas9-SaCas9 fusions. The SpCas9 protospacer is bold underlined and its PAM is in red, and the SaCas9 protospacer is double underlined and its PAM is green. (**b**) Lesion rates and types are determined by deep sequencing. Single nucleases generate small indels at their cognate cleavage sites, whereas dual nucleases (independent or fusion) may generate six types of lesion products. Note sgRNA4 is a suboptimal SaCas9 PAM with no activity on its own, but it produces precise deletions in the context of the SpCas9^{WT}-SaCas9^{WT} fusion. In many cases the overall SpCas9-SaCas9 fusion activity correlates with the SaCas9 activity at its target site. (**c**) Sequence information of the *VEGFA-TS3* target sites for SpCas9-SaCas9 fusions. The SpCas9 protospacer is bold underlined and its PAM is in red, and the SaCas9 protospacer is double underlined and its PAM is in green. (**d**) Lesion rates and types are determined by deep sequencing. Single nucleases generate small indels at their cognate cleavage sites, whereas dual nucleases (independent or fusion) may generate six types of lesion products. The red rectangle indicates the SaCas9 sgRNA that is used for specificity analysis of the SpCas9^{MT3}-dSaCas9 combination. Note sgRNA1 is a suboptimal D3 orientation, which is likely responsible for the absence of SpCas9^{MT3}-dSaCas9 fusion activity. For sgRNA2, the majority of the “imprecise deletions” (95%) are a sequence that contains a single “C” insertion between the two cleavage sites (a precise deletion with a 1 bp insertion), suggesting that one of the nucleases is not producing a blunt DSB in this context. Deep sequencing data are from three independent biological replicates performed on different days in HEK293T cells (**Supplementary Data 1**). Error bars indicate ±s.e.m.

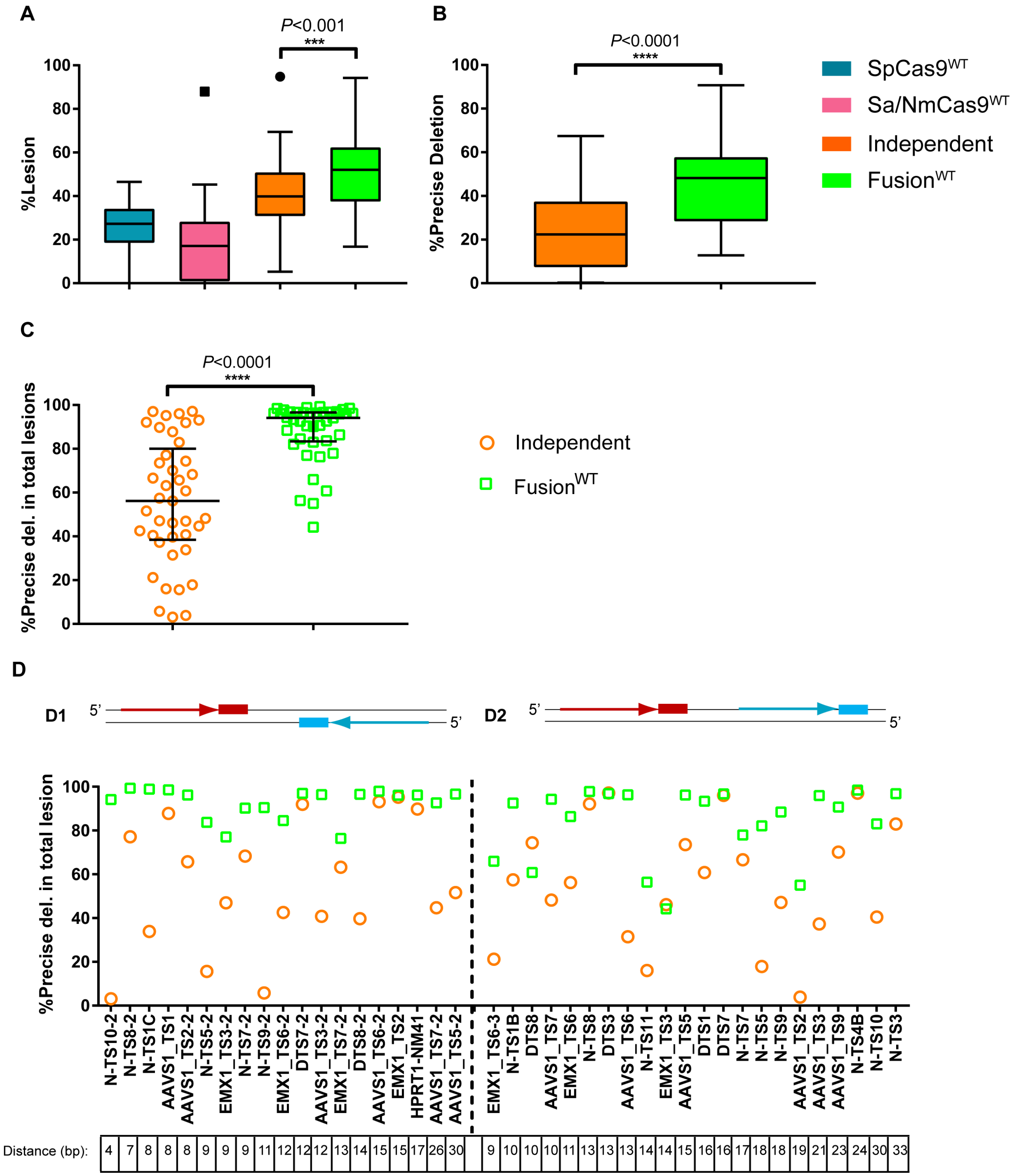
A
%Lesion
P<0.001
***
B
%Precise Deletion
P<0.0001
****
SpCas9WT
Sa/NmCas9WT
Independent
FusionWT
C
%Precise del. in total lesions
P<0.0001
****
Independent
FusionWT
D
D1
D2
5'
%Precise del. in total lesion
N-TS10-2
N-TS8-2
N-TS1C
AAVS1_TS1
AAVS1_TS2-2
N-TS5-2
EMX1_TS3-2
N-TS7-2
N-TS9-2
EMX1_TS6-2
DTS7-2
AAVS1_TS3-2
EMX1_TS7-2
DTS8-2
AAVS1_TS6-2
EMX1_TS2
HPRT1-NM41
AAVS1_TS7-2
AAVS1_TS5-2
EMX1_TS6-3
N-TS1B
DTS8
AAVS1_TS7
EMX1_TS6
N-TS8
DTS3
AAVS1_TS6
N-TS11
EMX1_TS3
AAVS1_TS5
DTS1
DTS7
N-TS7
N-TS5
N-TS9
AAVS1_TS2
AAVS1_TS3
AAVS1_TS9
N-TS4B
N-TS10
N-TS3
Distance (bp):
4 7 8 8 8 9 9 9 11 12 12 12 13 14 15 15 17 26 30
9 10 10 10 11 13 13 13 14 14 15 16 16 17 18 18 19 21 23 24 30 33

Supplementary Figure 5 | Activity profiles of SpCas9^{WT} (blue), Nm/SaCas9^{WT} (pink), SpCas9^{WT} + Nm/SaCas9^{WT} (orange), and SpCas9^{WT}-Nm/SaCas9^{WT} (green) nucleases at 41 genomic sites are determined by deep sequencing. (**a**) Total lesion rates for the given nucleases. Typically, SpCas9 is more active than Nm/SaCas9. Total lesion rates of the Cas9-Cas9 dual nucleases are higher than the monomeric Cas9s used in combination. (**b**) Cas9-Cas9 fusions generate higher rates of precise deletions in the target genome than two independent Cas9 monomers. Each Box plot is drawn by GraphPad Prism, where the box represents 25th and 75th percentile and the middle line is the median. Whiskers and outliers are defined by the Tukey method. (**c, d**) Cas9-Cas9 dual nucleases primarily generate exact deletion products whereas lesion types of the two independent monomeric Cas9s are site-dependent. (**c**) Overview of precise deletion levels generated by indicated nuclease platform. The scatter dot plot is drawn by GraphPad Prism, where the middle line indicates the median and the top and bottom lines border the interquartile ranges. (**d**) Aligned scatter plot for the comparison of precise deletion levels at each target site generated by indicated nuclease platform. Statistical significance is determined by one-way analysis of variance (ANOVA), "**" and "****" denote P <0.01 and <0.0001 respectively. Deep sequencing data are from single replicate in HEK293T cells (**Supplementary Data 1**).

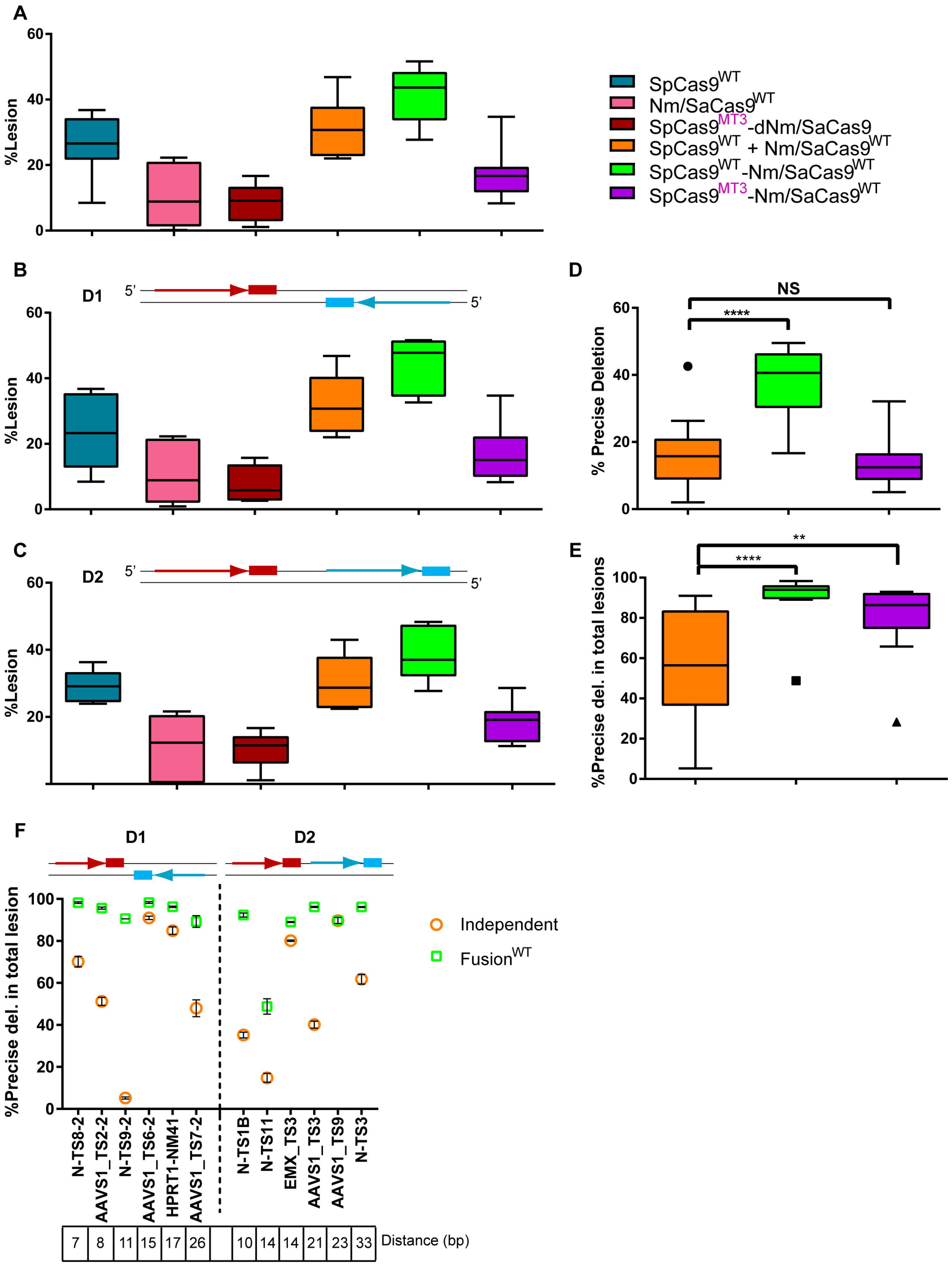
A
B
C
D
E
F
%Lesion
SpCas9WT
Nm/SaCas9WT
SpCas9MT3-dNm/SaCas9
SpCas9WT + Nm/SaCas9WT
SpCas9WT-Nm/SaCas9WT
SpCas9MT3-Nm/SaCas9WT
D1
D2
5’
NS
****
**
% Precise Deletion
%Precise del. in total lesions
%Precise del. in total lesion
Independent
FusionWT
N-TS8-2
AAVS1_TS2-2
N-TS9-2
AAVS1_TS6-2
HPRT1-NM41
AAVS1_TS7-2
N-TS1B
N-TS11
EMX_TS3
AAVS1_TS3
AAVS1_TS9
N-TS3
7 8 11 15 17 26 10 14 14 21 23 33
Distance (bp)

Supplementary Figure 6 | Activity profiles of SpCas9^{WT} (blue), Nm/SaCas9^{WT} (pink), SpCas9^{MT3}-dNm/SaCas9 (brown), SpCas9^{WT} + Nm/SaCas9^{WT} (orange), SpCas9^{WT}-Nm/SaCas9^{WT} (green), and SpCas9^{MT3}-Nm/SaCas9^{WT} (purple) nucleases at 12 genomic sites (6 D1 and 6 D2 configuration) are determined by deep sequencing. (**a**) Total lesion rates for the given nucleases at 12 genomic sites. (**b**) Total lesion rates for the given nucleases at 6 genomic sites with D1 configuration. (**c**). Total lesion rates for the given nucleases at 6 genomic sites with D2 configuration. (**d**) SpCas9^{WT}-Nm/SaCas9^{WT} fusions generate higher rates of precise deletions in the target genome than two independent Cas9 monomers. (**e**) Cas9-Cas9 dual nucleases primarily generate precise deletion products whereas lesion types of the two independent monomeric Cas9s are site-dependent. (**f**) Aligned scatter plot for the comparison of precise deletion levels at each target site generated by indicated nuclease platform. Each Box plot is drawn by GraphPad Prism, where the box represents 25th and 75th percentile and the middle line is the median. Whiskers and outliers are defined by the Tukey method. Statistical significance is determined by one-way analysis of variance (ANOVA), "**" and "****" denote, P <0.01 and P <0.0001 respectively. Deep sequencing data are from three independent biological replicates performed on different days in HEK293T cells (**Supplementary Data 1**). Error bars indicate ±s.e.m.

A

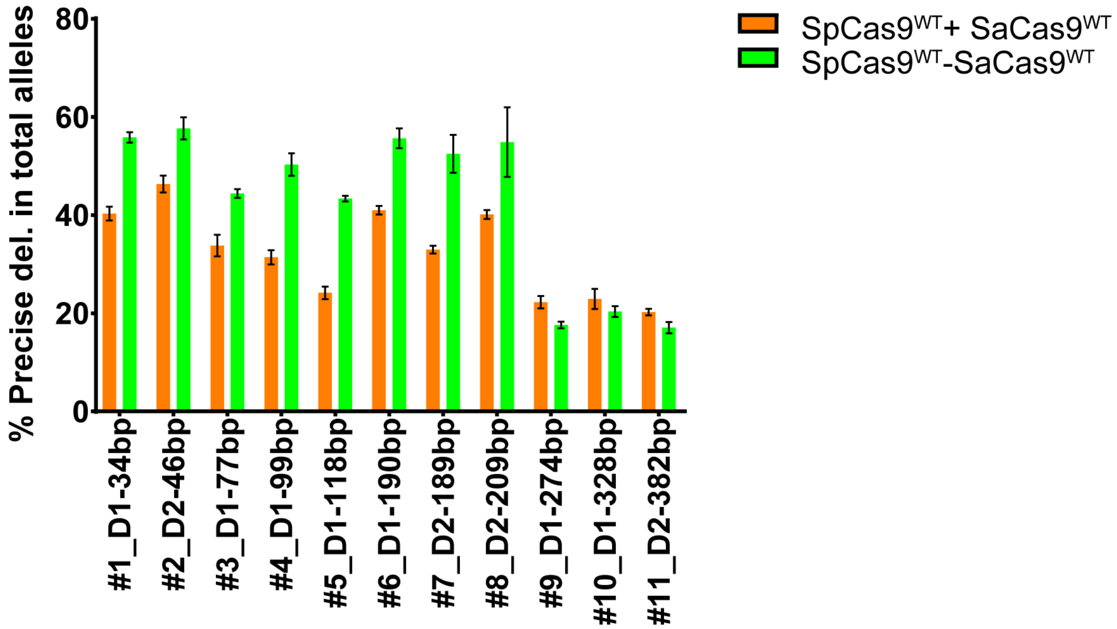

B

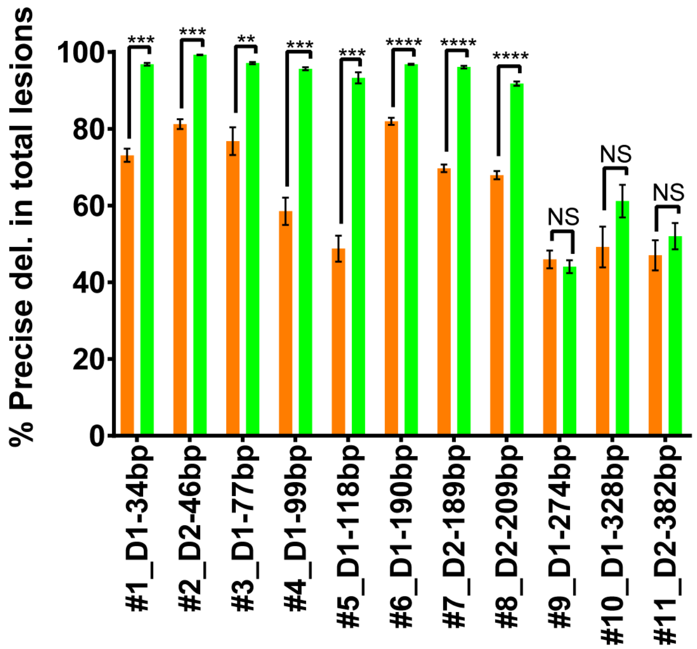

Supplementary Figure 7 | Activity profiles of dual nuclease platforms at the AAVS1 target sites listed in Supplementary Figure 2A. (**a**) SpCas9^{WT}-SaCas9^{WT} fusions generate higher rates of precise deletions in the target genome than two independent Cas9

monomers. (**b**) Cas9-Cas9 dual nucleases primarily generate precise deletion products at sites where protospacers are separated by less than 200 base pairs based on the fraction of all lesions that are precise deletions. Statistical significance between fusion and independent nucleases is determined by Student's t-test, "NS", "**", "***", and "****" denote not significant, P <0.01. P <0.001 and P <0.0001 respectively. Deep sequencing data are from three independent biological replicates performed on different days in HEK293T cells (**Supplementary Data 1**). Error bars indicate ±s.e.m.

A

```
NGC-1: GACATAACACACCAGGGTCAATACAACTTTGAAGCTAGTCTAGTGCAAGCTAACAGTTGCTTTTATCACAGGCTCCAGGAAGGGT
NGC-2: GACATAACACACCAGGGTCAATACAACTTTGAAGCTAGTCTAGTGCAAGCTAACAGTTGCTTTTATCACAGGCTCCAGGAAGGGT
NTG-1: GACATAACACACCAGGGTCAATACAACTTTGAAGCTAGTCTAGTGCAAGCTAACAGTTGCTTTTATCACAGGCTCCAGGAAGGGT
NTG-2: GACATAACACACCAGGGTCAATACAACTTTGAAGCTAGTCTAGTGCAAGCTAACAGTTGCTTTTATCACAGGCTCCAGGAAGGGT
NGT-1: GACATAACACACCAGGGTCAATACAACTTTGAAGCTAGTCTAGTGCAAGCTAACAGTTGCTTTTATCACAGGCTCCAGGAAGGGT
NGT-2: GACATAACACACCAGGGTCAATACAACTTTGAAGCTAGTCTAGTGCAAGCTAACAGTTGCTTTTATCACAGGCTCCAGGAAGGGT
NGA-1: GACATAACACACCAGGGTCAATACAACTTTGAAGCTAGTCTAGTGCAAGCTAACAGTTGCTTTTATCACAGGCTCCAGGAAGGGT
NCG-1: AGTCCTCTTCTACCCCACCCACGCCCCCACCCTAATCAGAGGCCAAACCCTTCCTGGAGCCTGTGATAAAAG
```

B

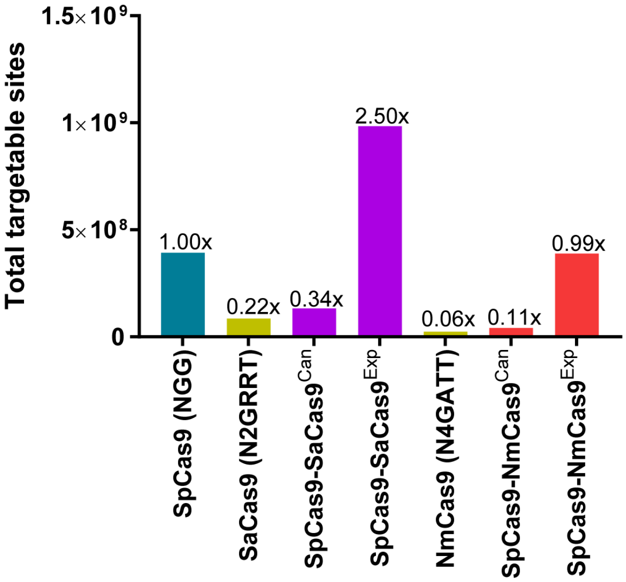

Supplementary Figure 8 | Cas9-Cas9 fusions allow expanded PAM usage. (**a**) Target site information; the SpCas9 protospacer is bold underlined and a suboptimal PAM element is in red, and the SaCas9 protospacer is double underlined and the cognate PAM element is green. (**b**) Computationally estimated total number of sites in the human genome that are targetable by each nuclease platform (**Supplementary Data 4**). For SpCas9-SaCas9 and SpCas9-NmCas9 fusions either canonical targets (Can) or expanded range (Exp) are listed, as defined in the main text.

*BCL11A*_enhancer+58

GATA-1 recognition site

```
GATA1_TS1:  ACCCACGCCCCCACCCTAATCAGAGGCCAAACCCTTCCTGGAGCCTGTGATAAAAGCAACTGTTAGCTT
GATA1_TS2:  ACCCACGCCCCCACCCTAATCAGAGGCCAAACCCTTCCTGGAGCCTGTGATAAAAGCAACTGTTAGCTTGCACTAGACTAGCTT
GATA1_TS3:  ACCCACGCCCCCACCCTAATCAGAGGCCAAACCCTTCCTGGAGCCTGTGATAAAAGCAACTGTTAGCTTGCACTAGACTAGCTT
GATA1_TS4:  ACCCACGCCCCCACCCTAATCAGAGGCCAAACCCTTCCTGGAGCCTGTGATAAAAGCAACTGTTAGCTTGCACTAGACTAGCTTC
GATA1_TS5:  ACCCACGCCCCCACCCTAATCAGAGGCCAAACCCTTCCTGGAGCCTGTGATAAAAGCAACTGTTAGCTTGCACTAGACTAGCTTCAAAGTTGTATTGACCCTGG
GATA1_TS6:  ACCCACGCCCCCACCCTAATCAGAGGCCAAACCCTTCCTGGAGCCTGTGATAAAAGCAACTGTTAGCTTGCACTAGACT
GATA1_TS7:  ACCCACGCCCCCACCCTAATCAGAGGCCAAACCCTTCCTGGAGCCTGTGATAAAAGCAACTGTTAGCTTGCACTAGACTAGC
GATA1_TS8:  ACCCACGCCCCCACCCTAATCAGAGGCCAAACCCTTCCTGGAGCCTGTGATAAAAGCAACTGTTAGCTTGCACTAGACTAGC
GATA1_TS9:  ACCCACGCCCCCACCCTAATCAGAGGCCAAACCCTTCCTGGAGCCTGTGATAAAAGCAACTGTTAGCTTGCACTAGACTAGCTTCAAAGTTGTATTGACCCTGG
GATA1_TS10: ACCCACGCCCCCACCCTAATCAGAGGCCAAACCCTTCCTGGAGCCTGTGATAAAAGCAACTGTTAGCTTGCACTAGACTAGC
GATA1_TS11: ACCCACGCCCCCACCCTAATCAGAGGCCAAACCCTTCCTGGAGCCTGTGATAAAAGCAACTGTTAGCTTGCACTAGACTAGC
GATA1_TS12: ACCCACGCCCCCACCCTAATCAGAGGCCAAACCCTTCCTGGAGCCTGTGATAAAAGCAACTGTTAGCTTGCACTAGACTAGC
```

Supplementary Figure 9 | Target site information of Cas9-Cas9 fusions for deletion of GATA1 binding element in the functional core of the *BCL11A* enhancer +58kb (highlighted in gray where the orange rectangle indicates the GATA1 binding site[1]). The SpCas9 protospacer is bold underlined with its PAM element in red, the SaCas9 protospacer is double underlined with its PAM element in green, and the NmCas9 protospacer is wavy underlined with its PAM element in blue.

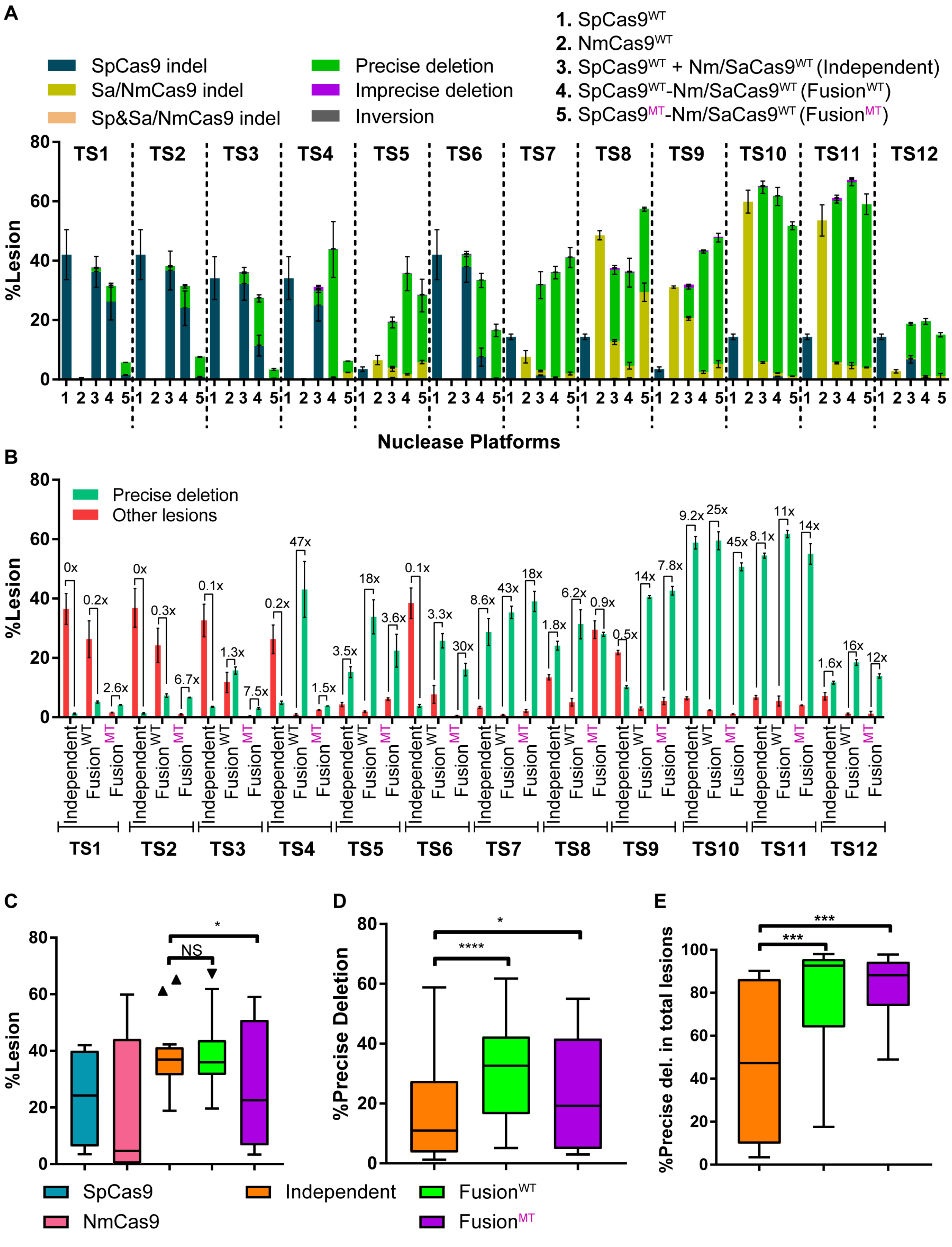
A
1. SpCas9WT
2. NmCas9WT
3. SpCas9WT + Nm/SaCas9WT (Independent)
4. SpCas9WT-Nm/SaCas9WT (FusionWT)
5. SpCas9MT-Nm/SaCas9WT (FusionMT)
SpCas9 indel
Sa/NmCas9 indel
Sp&Sa/NmCas9 indel
Precise deletion
Imprecise deletion
Inversion
%Lesion
TS1
TS2
TS3
TS4
TS5
TS6
TS7
TS8
TS9
TS10
TS11
TS12
Nuclease Platforms
B
Precise deletion
Other lesions
%Lesion
0x
0.2x
2.6x
0x
0.3x
6.7x
0.1x
1.3x
7.5x
0.2x
47x
1.5x
3.5x
18x
3.6x
0.1x
3.3x
30x
8.6x
43x
18x
1.8x
6.2x
0.9x
0.5x
14x
7.8x
9.2x
25x
45x
8.1x
11x
14x
1.6x
16x
12x
Independent
FusionWT
FusionMT
C
%Lesion
NS
*
D
%Precise Deletion
****
*
E
%Precise del. in total lesions
***
***
SpCas9
NmCas9
Independent
FusionWT
FusionMT

Supplementary Figure 10 | Assessment of the activity profiles of nucleases that are targeting the GATA1 binding element in *BCL11A* enhancer +58kb. (**a**) Lesion rates and types are determined by deep sequencing. Single nucleases generate small indels at their corresponding cleavage sites, whereas dual nucleases (independent, fusion^{WT}, or fusion^{MT}) may generate six types of lesion products. (**b**) Ratios of precise deletions relative to other types of lesions for each nuclease and target site. (**c**, **d**, **e**) Overview of the nuclease activities at 12 GATA1 target sites: (**c**) Total lesion rates, (**d**) precise deletion rates, and (**e**) fraction of precise deletions among all lesions. Each Box plot is drawn by GraphPad Prism, where the box represents 25th and 75th percentile and the middle line is the median. Whiskers and outliers are defined by the Tukey method. Statistical significance is determined by one-way analysis of variance (ANOVA), “*”, “***” and “****” denote P <0.05, P <0.001 and P <0.0001 respectively. Deep sequencing data are from three independent biological replicates performed on different days in HEK293T cells (**Supplementary Data 1**). Error bars indicate ±s.e.m.

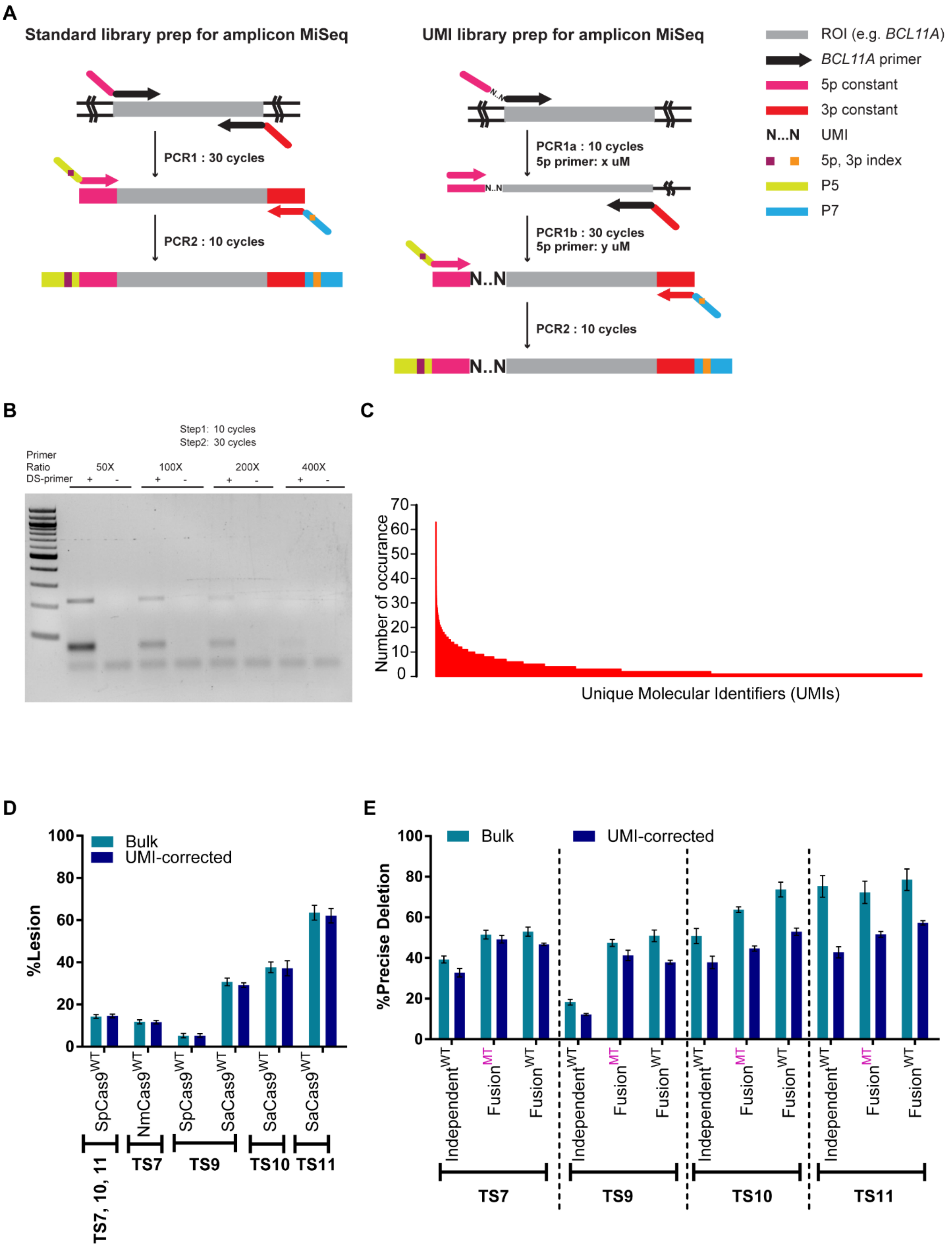
A
Standard library prep for amplicon MiSeq
PCR1 : 30 cycles
PCR2 : 10 cycles
UMI library prep for amplicon MiSeq
PCR1a : 10 cycles
5p primer: x uM
PCR1b : 30 cycles
5p primer: y uM
PCR2 : 10 cycles
N..N
ROI (e.g. BCL11A)
BCL11A primer
5p constant
3p constant
N...N UMI
5p, 3p index
P5
P7
B
Step1: 10 cycles
Step2: 30 cycles
Primer Ratio
50X
100X
200X
400X
DS-primer
C
Number of occurance
Unique Molecular Identifiers (UMIs)
D
%Lesion
Bulk
UMI-corrected
SpCas9WT
NmCas9WT
SpCas9WT
SaCas9WT
SaCas9WT
SaCas9WT
TS7, 10, 11
TS7
TS9
TS10
TS11
E
%Precise Deletion
Bulk
UMI-corrected
IndependentWT
FusionMT
FusionWT
TS7
TS9
TS10
TS11

Supplementary Figure 11 | Accurate quantification of the segmental deletions via unique molecular identifiers (UMI) and linear amplification mediated (LAM) PCR. (**a**) Schematic of the library construction process for deep sequencing. Left, standard library prep where the region of interest (gray box) is first amplified with locus-specific oligos (black arrows) bearing constant sequences (magenta and red) for TruSeq adaptor recognition. Next, the amplicon is further amplified from the constant sequences with oligos bearing indexes (purple and orange squares) and overhangs for P7 (light green) and P5 (blue). Right, library construction for UMI-correction, where the genomic DNA is pre-amplified with oligos bearing UMI (N…N) and 5' constant sequence (magenta) via LAM PCR. Next, the single-stranded DNA is amplified with 5' constant sequence and 3' locus specific primer (black arrow) bearing 3' constant sequence (red). Then, the amplicon is further amplified from the constant sequences with oligos bearing indexes (purple and orange squares) and overhangs for P7 (light green) and P5 (blue). (**b**) An example of an agarose gel electrophoresis picture showing the outcome of PCR 1b of the UMI-correction library. The primer ratio indicates the value of 5' constant primer concentration (y µM) divided by 5' BCL11A_enh58_UMI primer concentration (x µM). In the absence of the 5' constant primer (DS-primer), the amplification product is not visible. (**c**) A representative histogram plotting the distribution of particular UMI abundance of TS11_SpCas9^{WT}-SaCas9^{WT}_Replicate-3 sample in the deep sequencing library. (**d**) For single nucleases the analysis of total lesions indicate that indels do not generate amplification bias. (**e**) For dual nucleases, the rate of precise deletions are over-estimated via bulk analysis. Deep sequencing data are from three independent biological replicates performed on different days in HEK293T cells (**Supplementary Data 1**). Error bars indicate ±s.e.m.

*BCL11A*_enhancer+58

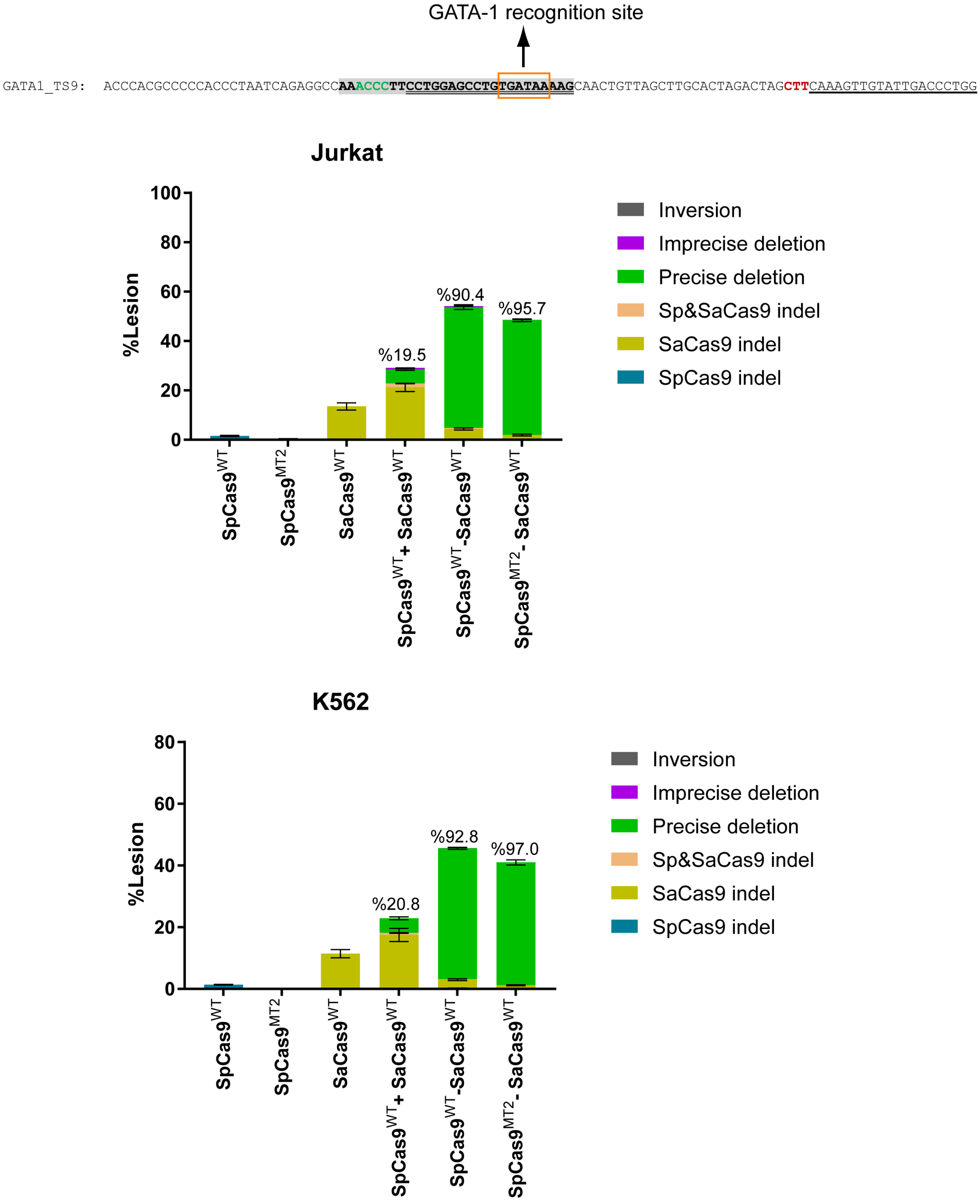

Supplementary Figure 12 | Cas9-Cas9 fusions display similar nuclease activity at BCL11A locus GATA1-TS9 in different cell lines. Activity profiles of various nucleases in Jurkat (top) and K562 (bottom) cells. Lesion rates and types are determined by deep sequencing. Single nucleases generate small indels at their cognate cleavage sites, whereas dual nucleases (independent or fusion) may generate six types of lesion products. Deep sequencing data are from three independent biological replicates with UMI correction performed on different days (**Supplementary Data 1**). Error bars indicate ±s.e.m.

Supplementary References:

1. Canver, M.C. et al. BCL11A enhancer dissection by Cas9-mediated in situ saturating mutagenesis. *Nature* **527**, 192-197 (2015).
