# Supplementary material for: Orthogonal Cas9–Cas9 chimeras provide a versatile platform for genome editing
Source: Nat Commun. 2018 Nov 19;9:4856. doi: 10.1038/s41467-018-07310-x (PMC6242970; doi:10.1038/s41467-018-07310-x)
Supplement: Supplementary file 3 — Description of Additional Supplementary Files [file 41467_2018_7310_MOESM3_ESM.pdf]

DESCRIPTION OF ADDITIONAL SUPPLEMENTARY FILES

File Name: Supplementary Data 1
Description: Overview of the PCR amplicon deep sequencing datasets. There are multiple tabs for experiments relating to VEGFA-TS2, VEGFA-TS3, VEGFA-TS3 off-targets, 41 genomic sites, 12 genomic sites, AAVS1 spacing data (SpCas9MTdSaCas9), SpCas9 suboptimal PAMs, BCL11A enhancer +58kb 12 sites, BCL11A enhancer +58kb 4 sites with UMI, BCL11A enhancer +58kb in Jurkat and K562 cells, BCL11A enhancer +58kb 4 sites off-targets, and AAVS1 large deletion (SpCas9WT-SaCas9WT). Each tab contains information regarding the MiSeq run, sample name, and index sequence. “Stacked bar graphs” tab reports overall analyzed data used to generate main figure stacked bar graphs. The final tab reports the statistical data.

File Name: Supplementary Data 2
Description: Overview of the GUIDE-seq datasets.

File Name: Supplementary Data 3
Description: Sequence information of the target sites, off-target sites and the primers used in amplicon deep sequencing experiments.

File Name: Supplementary Data 4
Description: Estimated number of target sites for SpCas9- NmCas9 and SpCas9-SaCas9 fusions within each chromosome of the human genome.

File Name: Supplementary Data 5
Description: Annotated protein sequences of key Cas9-Cas9 constructs utilized in this study.
