# Supplementary material for: Orthogonal Cas9–Cas9 chimeras provide a versatile platform for genome editing
Source: Nat Commun. 2018 Nov 19;9:4856. doi: 10.1038/s41467-018-07310-x (PMC6242970; doi:10.1038/s41467-018-07310-x)
Supplement: Supplementary file 8 — Supplementary Data 5 [file 41467_2018_7310_MOESM8_ESM.pdf]

Supplementary Data 5 | Protein sequences of the Cas9-Cas9 fusions used in this study.

>**SpCas9**^{MT3}-dNmCas9

```
MKRPAATKKAGQAKKKKLDGSGSPKKKRKVPWDKKYSIGLDIGTNSVGWAVITDEYKVPSKKFKVLGNTD
RHSIKKNLIGALLFDSGETAEATRLKRTARRRYTRRKNRICYLQEIFSNEMAKVDDSFFHRLEESFLVEE
DKKHERHPIFGNIVDEVAYHEKYPTIYHLRKKLVDSTDKADLRLIYLALAHMIKFRGHFLIEGDLNPDNS
DVDKLFIQLVQTYNQLFEENPINASGVDAKAILSARLSKSRRLENLIAQLPGEKKNGLFGNLIALSLGLT
PNFKSNFDLAEDAKLQLSKDTYDDDLDNLLAQIGDQYADLFLAAKNLSDAILLSDILRVNTEITKAPLSA
SMIKRYDEHHQDLTLLKALVRQQLPEKYKEIFFDQSKNGYAGYIDGGASQEEFYKFIKPILEKMDGTEEL
LVKLNREDLLRKQRTFDNGSIPHQIHLGELHAILRRQEDFYPFLKDNREKIEKILTFRIPYYVGPLARGN
SRFAWMTRKSEETITPWNFEEVVDKGASAQSFIERMTNFDKNLPNEKVLPKHSLLYEYFTVYNELTKVKY
VTEGMRKPAFLSGEQKKAIVDLLFKTNRKVTVKQLKEDYFKKIECFDSVEISGVEDRFNASLGTYHDLLK
IIKDKDFLDNEENEDILEDIVLTLTLFEDREMIEERLKTYAHLFDDKVMKQLKRRRYTGWGRLSRKLING
IRDKQSGKTILDFLKSDGFANRNFMQLIHDDSLTFKEDIQKAQVSGQGDSLHEHIANLAGSPAIKKGILQ
TVKVVDELVKVMGRHKPENIVIEMARENQTTQKGQKNSRERMKRIEEGIKELGSQILKEHPVENTQLQNE
KLYLYYLQNGRDMYVDQELDINRLSDYDVDHIVPQSFLKDDSIDNKVLTRSDKNRGKSDNVPSEEVVKKM
KNYWRQLLNAKLITQRKFDNLTKAERGGLSELDKAGFIKRQLVETRQITKHVAQILDSRMNTKYDENDKL
IREVKVITLKSKLVSDFRKDFQFYKVREINNYHHAHDAYLNAVVGTALIKKYPKLESEFVYGDYKVYDVR
KMIAKSEQEIGKATAKYFFYSNIMNFFKTEITLANGEIRKRPLIETNGETGEIVWDKGRDFATVRKVLSM
PQVNIVKKTEVQTGGFSKESILPKRNSDKLIARKKDWDPKKYGGFDSPTVAYSVLVVAKVEKGKSKKLKS
VKELLGITIMERSSFEKNPIDFLEAKGYKEVKKDLIIKLPKYSLFELENGRKRMLASAGELQKGNELALP
SKYVNFLYLASHYEKLKGSPEDNEQKQLFVEQHKHYLDEIIEQISEFSKRVILADANLDKVLSAYNKHRD
KPIREQAENIIHLFTLTNLGAPAAFKYFDTTIDRKKYTSTKEVLDATLIHQSITGLYETRIDLSQLGGDG
TGGPKKKRKVYPYDVPDYAGYPYDVPDYAGSYPYDVPDYAGSAAPAAKKKKLDFESGMPKKKRKVAAFKP
NPINYILGLAIGIASVGWAMVEIDEDENPICLIDLGVRVFERAEVPKTGDSLAMARRLARSVRRLTRRRA
HRLLRARRLLKREGVLQAADFDENGLIKSLPNTPWQLRAAALDRKLTPLEWSAVLLHLIKHRGYLSQRKN
EGETADKELGALLKGVADNAHALQTGDFRTPAELALNKFEKESGHIRNQRGDYSHTFSRKDLQAELILLF
EKQKEFGNPHVSGGLKEGIETLLMTQRPALSGDAVQKMLGHCTFEPAEPKAAKNTYTAERFIWLTKLNNL
RILEQGSERPLTDTERATLMDEPYRKSKLTYAQARKLLGLEDTAFFKGLRYGKDNAEASTLMEMKAYHAI
SRALEKEGLKDKKSPLNLSPELQDEIGTAFSLFKTDEDITGRLKDRIQPEILEALLKHISFDKFVQISLK
ALRRIVPLMEQGKRYDEACAEIYGDHYGKKNTEEKIYLPPIPADEIRNPVVLRALSQARKVINGVVRRYG
SPARIHIETAREVGKSFKDRKEIEKRQEENRKDREKAAAKFREYFPNFVGEPKSKDILKLRLYEQQHGKC
LYSGKEINLGRLNEKGYVEIAAALPFSRTWDDSFNNKVLVLGSEAQNKGNQTPYEYFNGKDNSREWQEFK
ARVETSRFPRSKKQRILLQKFDEDGFKERNLNDTRYVNRFLCQFVADRMRLTGKGKKRVFASNGQITNLL
RGFWGLRKVRAENDRHHALDAVVVACSTVAMQQKITRFVRYKEMNAFDGKTIDKETGEVLHQKTHFPQPW
EFFAQEVMIRVFGKPDGKPEFEEADTPEKLRTLLAEKLSSRPEAVHEYVTPLFVSRAPNRKMSGQGHMET
VKSAKRLDEGVSVLRVPLTQLKLKDLEKMVNREREPKLYEALKARLEAHKDDPAKAFAEPFYKYDKAGNR
TQQVKAVRVEQVQKTGVWVRNHNGIADNATMVRVDVFEKGDKYYLVPIYSWQVAKGILPDRAVVQGKDEE
DWQLIDDSFNFKFSLHPNDLVEVITKKARMFGYFASCHRGTGNINIRIHDLDHKIGKNGILEGIGVKTAL
SFQKYQIDELGKEIRPCRLKKRPPVRSRADPKKKRKVEASLEKRPAATKKAGQAKKKKGS
```

>SpCas9^{WT}-NmCas9^{WT}

MKRPAATKKAGQAKKKKLDGSGSPKKKRKVPWDKKYSIGLDIGTNSVGWAVITDEYKVPSKKFKVLGNTD
RHSIKKNLIGALLFDSGETAEATRLKRTARRRYTRRKNRICYLQEIFSNEMAKVDDSFFHRLEESFLVEE
DKKHERHPIFGNIVDEVAYHEKYPTIYHLRKKLVDSTDKADLRLIYLALAHMIKFRGHFLIEGDLNPDNS
DVDKLFIQLVQTYNQLFEENPINASGVDAKAILSARLSKSRRLENLIAQLPGEKKNGLFGNLIALSLGLT
PNFKSNFDLAEDAKLQLSKDTYDDDLDNLLAQIGDQYADLFLAAKNLSDAILLSDILRVNTEITKAPLSA
SMIKRYDEHHQDLTLLKALVRQQLPEKYKEIFFDQSKNGYAGYIDGGASQEEFYKFIKPILEKMDGTEEL
LVKLNREDLLRKQRTFDNGSIPHQIHLGELHAILRRQEDFYPFLKDNREKIEKILTFRIPYYVGPLARGN
SRFAWMTRKSEETITPWNFEEVVDKGASAQSFIERMTNFDKNLPNEKVLPKHSLLYEYFTVYNELTKVKY
VTEGMRKPAFLSGEQKKAIVDLLFKTNRKVTVKQLKEDYFKKIECFDSVEISGVEDRFNASLGTYHDLLK
IIKDKDFLDNEENEDILEDIVLTLTLFEDREMIEERLKTYAHLFDDKVMKQLKRRRYTGWGRLSRKLING
IRDKQSGKTILDFLKSDGFANRNFMQLIHDDSLTFKEDIQKAQVSGQGDSLHEHIANLAGSPAIKKGILQ
TVKVVDELVKVMGRHKPENIVIEMARENQTTQKGQKNSRERMKRIEEGIKELGSQILKEHPVENTQLQNE
KLYLYYLQNGRDMYVDQELDINRLSDYDVDHIVPQSFLKDDSIDNKVLTRSDKNRGKSDNVPSEEVVKKM
KNYWRQLLNAKLITQRKFDNLTKAERGGLSELDKAGFIKRQLVETRQITKHVAQILDSRMNTKYDENDKL
IREVKVITLKSKLVSDFRKDFQFYKVREINNYHHAHDAYLNAVVGTALIKKYPKLESEFVYGDYKVYDVR
KMIAKSEQEIGKATAKYFFYSNIMNFFKTEITLANGEIRKRPLIETNGETGEIVWDKGRDFATVRKVLSM
PQVNIVKKTEVQTGGFSKESILPKRNSDKLIARKKDWDPKKYGGFDSPTVAYSVLVVAKVEKGKSKKLKS
VKELLGITIMERSSFEKNPIDFLEAKGYKEVKKDLIIKLPKYSLFELENGRKRMLASAGELQKGNELALP
SKYVNFLYLASHYEKLKGSPEDNEQKQLFVEQHKHYLDEIIEQISEFSKRVILADANLDKVLSAYNKHRD
KPIREQAENIIHLFTLTNLGAPAAFKYFDTTIDRKRYTSTKEVLDATLIHQSITGLYETRIDLSQLGGDG
TGGPKKKRKVYPYDVPDYAGYPYDVPDYAGSYPYDVPDYAGSAAPAAKKKKLDFESGMPKKKRKVAAFKP
NPINYILGL**D**IGIASVGWAMVEIDEDENPICLIDLGVRVFERAEVPKTGDSLAMARRLARSVRRLTRRRA
HRLLRARRLLKREGVLQAADFDENGLIKSLPNTPWQLRAAALDRKLTPLEWSAVLLHLIKHRGYLSQRKN
EGETADKELGALLKGVADNAHALQTGDFRTPAELALNKFEKESGHIRNQRGDYSHTFSRKDLQAELILLF
EKQKEFGNPHVSGGLKEGIETLLMTQRPALSGDAVQKMLGHCTFEPAEPKAAKNTYTAERFIWLTKLNNL
RILEQGSERPLTDTERATLMDEPYRKSKLTYAQARKLLGLEDTAFFKGLRYGKDNAEASTLMEMKAYHAI
SRALEKEGLKDKKSPLNLSPELQDEIGTAFSLFKTDEDITGRLKDRIQPEILEALLKHISFDKFVQISLK
ALRRIVPLMEQGKRYDEACAEIYGDHYGKKNTEEKIYLPPIPADEIRNPVVLRALSQARKVINGVVRRYG
SPARIHIETAREVGKSFKDRKEIEKRQEENRKDREKAAAKFREYFPNFVGEPKSKDILKLRLYEQQHGKC
LYSGKEINLGRLNEKGYVEI**DH**ALPFSRTWDDSFNNKVLVLGSE**N**QNKGNQTPYEYFNGKDNSREWQEFK
ARVETSRFPRSKKQRILLQKFDEDGFKERNLNDTRYVNRFLCQFVADRMRLTGKGKKRVFASNGQITNLL
RGFWGLRKVRAENDRHHALDAVVVACSTVAMQQKITRFVRYKEMNAFDGKTIDKETGEVLHQKTHFPQPW
EFFAQEVMIRVFGKPDGKPEFEEADTPEKLRTLLAEKLSSRPEAVHEYVTPLFVSRAPNRKMSGQGHMET
VKSAKRLDEGVSVLRVPLTQLKLKDLEKMVNREREPKLYEALKARLEAHKDDPAKAFAEPFYKYDKAGNR
TQQVKAVRVEQVQKTGVWVRNHNGIADNATMVRVDVFEKGDKYYLVPIYSWQVAKGILPDRAVVQGKDEE
DWQLIDDSFNFKFSLHPNDLVEVITKKARMFGYFASCHRGTGNINIRIHDLDHKIGKNGILEGIGVKTAL
SFQKYQIDELGKEIRPCRLKKRPPVRSRADPKKKRKVEASLEKRPAATKKAGQAKKKKGS

>SpCas9^{MT3}-NmCas9^{WT}

MKRPAATKKAGQAKKKKLDGSGSPKKKRKVPWDKKYSIGLDIGTNSVGWAVITDEYKVPSKKFKVLGNTD
RHSIKKNLIGALLFDSGETAEATRLKRTARRRYTRRKNRICYLQEIFSNEMAKVDDSFFHRLEESFLVEE
DKKHERHPIFGNIVDEVAYHEKYPTIYHLRKKLVDSTDKADLRLIYLALAHMIKFRGHFLIEGDLNPDNS
DVDKLFIQLVQTYNQLFEENPINASGVDAKAILSARLSKSRRLENLIAQLPGEKKNGLFGNLIALSLGLT
PNFKSNFDLAEDAKLQLSKDTYDDDLDNLLAQIGDQYADLFLAAKNLSDAILLSDILRVNTEITKAPLSA
SMIKRYDEHHQDLTLLKALVRQQLPEKYKEIFFDQSKNGYAGYIDGGASQEEFYKFIKPILEKMDGTEEL
LVKLNREDLLRKQRTFDNGSIPHQIHLGELHAILRRQEDFYPFLKDNREKIEKILTFRIPYYVGPLARGN
SRFAWMTRKSEETITPWNFEEVVDKGASAQSFIERMTNFDKNLPNEKVLPKHSLLYEYFTVYNELTKVKY
VTEGMRKPAFLSGEQKKAIVDLLFKTNRKVTVKQLKEDYFKKIECFDSVEISGVEDRFNASLGTYHDLLK
IIKDKDFLDNEENEDILEDIVLTLTLFEDREMIEERLKTYAHLFDDKVMKQLKRRRYTGWGRLSRKLING
IRDKQSGKTILDFLKSDGFANRNFMQLIHDDSLTFKEDIQKAQVSGQGDSLHEHIANLAGSPAIKKGILQ
TVKVVDELVKVMGRHKPENIVIEMARENQTTQKGQKNSRERMKRIEEGIKELGSQILKEHPVENTQLQNE
KLYLYYLQNGRDMYVDQELDINRLSDYDVDHIVPQSFLKDDSIDNKVLTRSDKNRGKSDNVPSEEVVKKM
KNYWRQLLNAKLITQRKFDNLTKAERGGLSELDKAGFIKRQLVETRQITKHVAQILDSRMNTKYDENDKL
IREVKVITLKSKLVSDFRKDFQFYKVREINNYHHAHDAYLNAVVGTALIKKYPKLESEFVYGDYKVYDVR
KMIAKSEQEIGKATAKYFFYSNIMNFFKTEITLANGEIRKRPLIETNGETGEIVWDKGRDFATVRKVLSM
PQVNIVKKTEVQTGGFSKESILPKRNSDKLIARKKDWDPKKYGGFDSPTVAYSVLVVAKVEKGKSKKLKS
VKELLGITIMERSSFEKNPIDFLEAKGYKEVKKDLIIKLPKYSLFELENGRKRMLASAGELQKGNELALP
SKYVNFLYLASHYEKLKGSPEDNEQKQLFVEQHKHYLDEIIEQISEFSKRVILADANLDKVLSAYNKHRD
KPIREQAENIIHLFTLTNLGAPAAFKYFDTTIDRKKYTSTKEVLDATLIHQSITGLYETRIDLSQLGGDG
TGGPKKKRKVYPYDVPDYAGYPYDVPDYAGSYPYDVPDYAGSAAPAAKKKKLDFESGMPKKKRKVAAFKP
NPINYILGL**D**IGIASVGWAMVEIDEDENPICLIDLGVRVFERAEVPKTGDSLAMARRLARSVRRLTRRRA
HRLLRARRLLKREGVLQAADFDENGLIKSLPNTPWQLRAAALDRKLTPLEWSAVLLHLIKHRGYLSQRKN
EGETADKELGALLKGVADNAHALQTGDFRTPAELALNKFEKESGHIRNQRGDYSHTFSRKDLQAELILLF
EKQKEFGNPHVSGGLKEGIETLLMTQRPALSGDAVQKMLGHCTFEPAEPKAAKNTYTAERFIWLTKLNNL
RILEQGSERPLTDTERATLMDEPYRKSKLTYAQARKLLGLEDTAFFKGLRYGKDNAEASTLMEMKAYHAI
SRALEKEGLKDKKSPLNLSPELQDEIGTAFSLFKTDEDITGRLKDRIQPEILEALLKHISFDKFVQISLK
ALRRIVPLMEQGKRYDEACAEIYGDHYGKKNTEEKIYLPPIPADEIRNPVVLRALSQARKVINGVVRRYG
SPARIHIETAREVGKSFKDRKEIEKRQEENRKDREKAAAKFREYFPNFVGEPKSKDILKLRLYEQQHGKC
LYSGKEINLGRLNEKGYVEI**DH**ALPFSRTWDDSFNNKVLVLGSE**N**QNKGNQTPYEYFNGKDNSREWQEFK
ARVETSRFPRSKKQRILLQKFDEDGFKERNLNDTRYVNRFLCQFVADRMRLTGKGKKRVFASNGQITNLL
RGFWGLRKVRAENDRHHALDAVVVACSTVAMQQKITRFVRYKEMNAFDGKTIDKETGEVLHQKTHFPQPW
EFFAQEVMIRVFGKPDGKPEFEEADTPEKLRTLLAEKLSSRPEAVHEYVTPLFVSRAPNRKMSGQGHMET
VKSAKRLDEGVSVLRVPLTQLKLKDLEKMVNREREPKLYEALKARLEAHKDDPAKAFAEPFYKYDKAGNR
TQQVKAVRVEQVQKTGVWVRNHNGIADNATMVRVDVFEKGDKYYLVPIYSWQVAKGILPDRAVVQGKDEE
DWQLIDDSFNFKFSLHPNDLVEVITKKARMFGYFASCHRGTGNINIRIHDLDHKIGKNGILEGIGVKTAL
SFQKYQIDELGKEIRPCRLKKRPPVRSRADPKKKRKVEASLEKRPAATKKAGQAKKKKGS

>SpCas9^{MT2}-NmCas9^{WT}

MKRPAATKKAGQAKKKKLDGSGSPKKKRKVPWDKKYSIGLDIGTNSVGWAVITDEYKVPSKKFKVLGNTD
RHSIKKNLIGALLFDSGETAEATRLKRTARRRYTRRKNRICYLQEIFSNEMAKVDDSFFHRLEESFLVEE
DKKHERHPIFGNIVDEVAYHEKYPTIYHLRKKLVDSTDKADLRLIYLALAHMIKFRGHFLIEGDLNPDNS
DVDKLFIQLVQTYNQLFEENPINASGVDAKAILSARLSKSRRLENLIAQLPGEKKNGLFGNLIALSLGLT
PNFKSNFDLAEDAKLQLSKDTYDDDLDNLLAQIGDQYADLFLAAKNLSDAILLSDILRVNTEITKAPLSA
SMIKRYDEHHQDLTLLKALVRQQLPEKYKEIFFDQSKNGYAGYIDGGASQEEFYKFIKPILEKMDGTEEL
LVKLNREDLLRKQRTFDNGSIPHQIHLGELHAILRRQEDFYPFLKDNREKIEKILTFRIPYYVGPLARGN
SRFAWMTRKSEETITPWNFEEVVDKGASAQSFIERMTNFDKNLPNEKVLPKHSLLYEYFTVYNELTKVKY
VTEGMRKPAFLSGEQKKAIVDLLFKTNRKVTVKQLKEDYFKKIECFDSVEISGVEDRFNASLGTYHDLLK
IIKDKDFLDNEENEDILEDIVLTLTLFEDREMIEERLKTYAHLFDDKVMKQLKRRRYTGWGRLSRKLING
IRDKQSGKTILDFLKSDGFANRNFMQLIHDDSLTFKEDIQKAQVSGQGDSLHEHIANLAGSPAIKKGILQ
TVKVVDELVKVMGRHKPENIVIEMARENQTTQKGQKNSRERMKRIEEGIKELGSQILKEHPVENTQLQNE
KLYLYYLQNGRDMYVDQELDINRLSDYDVDHIVPQSFLKDDSIDNKVLTRSDKNRGKSDNVPSEEVVKKM
KNYWRQLLNAKLITQRKFDNLTKAERGGLSELDKAGFIKRQLVETRQITKHVAQILDSRMNTKYDENDKL
IREVKVITLKSKLVSDFRKDFQFYKVREINNYHHAHDAYLNAVVGTALIKKYPKLESEFVYGDYKVYDVR
KMIAKSEQEIGKATAKYFFYSNIMNFFKTEITLANGEIRKRPLIETNGETGEIVWDKGRDFATVRKVLSM
PQVNIVKKTEVQTGGFSKESILPKRNSDKLIARKKDWDPKKYGGFDSPTVAYSVLVVAKVEKGKSKKLKS
VKELLGITIMERSSFEKNPIDFLEAKGYKEVKKDLIIKLPKYSLFELENGRKRMLASAGELQKGNELALP
SKYVNFLYLASHYEKLKGSPEDNEQKQLFVEQHKHYLDEIIEQISEFSKRVILADANLDKVLSAYNKHRD
KPIREQAENIIHLFTLTNLGAPAAFKYFDTTIDSKRYTSTKEVLDATLIHQSITGLYETRIDLSQLGGDG
TGGPKKKRKVYPYDVPDYAGYPYDVPDYAGSYPYDVPDYAGSAAPAAKKKKLDFESGMPKKKRKVAAFKP
NPINYILGL**D**IGIASVGWAMVEIDEDENPICLIDLGVRVFERAEVPKTGDSLAMARRLARSVRRLTRRRA
HRLLRARRLLKREGVLQAADFDENGLIKSLPNTPWQLRAAALDRKLTPLEWSAVLLHLIKHRGYLSQRKN
EGETADKELGALLKGVADNAHALQTGDFRTPAELALNKFEKESGHIRNQRGDYSHTFSRKDLQAELILLF
EKQKEFGNPHVSGGLKEGIETLLMTQRPALSGDAVQKMLGHCTFEPAEPKAAKNTYTAERFIWLTKLNNL
RILEQGSERPLTDTERATLMDEPYRKSKLTYAQARKLLGLEDTAFFKGLRYGKDNAEASTLMEMKAYHAI
SRALEKEGLKDKKSPLNLSPELQDEIGTAFSLFKTDEDITGRLKDRIQPEILEALLKHISFDKFVQISLK
ALRRIVPLMEQGKRYDEACAEIYGDHYGKKNTEEKIYLPPIPADEIRNPVVLRALSQARKVINGVVRRYG
SPARIHIETAREVGKSFKDRKEIEKRQEENRKDREKAAAKFREYFPNFVGEPKSKDILKLRLYEQQHGKC
LYSGKEINLGRLNEKGYVEI**DH**ALPFSRTWDDSFNNKVLVLGSE**N**QNKGNQTPYEYFNGKDNSREWQEFK
ARVETSRFPRSKKQRILLQKFDEDGFKERNLNDTRYVNRFLCQFVADRMRLTGKGKKRVFASNGQITNLL
RGFWGLRKVRAENDRHHALDAVVVACSTVAMQQKITRFVRYKEMNAFDGKTIDKETGEVLHQKTHFPQPW
EFFAQEVMIRVFGKPDGKPEFEEADTPEKLRTLLAEKLSSRPEAVHEYVTPLFVSRAPNRKMSGQGHMET
VKSAKRLDEGVSVLRVPLTQLKLKDLEKMVNREREPKLYEALKARLEAHKDDPAKAFAEPFYKYDKAGNR
TQQVKAVRVEQVQKTGVWVRNHNGIADNATMVRVDVFEKGDKYYLVPIYSWQVAKGILPDRAVVQGKDEE
DWQLIDDSFNFKFSLHPNDLVEVITKKARMFGYFASCHRGTGNINIRIHDLDHKIGKNGILEGIGVKTAL
SFQKYQIDELGKEIRPCRLKKRPPVRSRADPKKKRKVEASLEKRPAATKKAGQAKKKKGS

>**SpCas9**^{WT}-dNmCas9

M**KRPAATKKAGQAKKKK**LDGSGS**PKKKRKV**PW**DKKYSIGLDIGTNSVGWAVITDEYKVPSKKFKVLGNTD**
RHSIKKNLIGALLFDSGETAEATRLKRTARRRYTRRKNRICYLQEIFSNEMAKVDDSFFHRLEESFLVEE
DKKHERHPIFGNIVDEVAYHEKYPTIYHLRKKLVDSTDKADLRLIYLALAHMIKFRGHFLIEGDLNPDNS
DVDKLFIQLVQTYNQLFEENPINASGVDAKAILSARLSKSRRLENLIAQLPGEKKNGLFGNLIALSLGLT
PNFKSNFDLAEDAKLQLSKDTYDDDLDNLLAQIGDQYADLFLAAKNLSDAILLSDILRVNTEITKAPLSA
SMIKRYDEHHQDLTLLKALVRQQLPEKYKEIFFDQSKNGYAGYIDGGASQEEFYKFIKPILEKMDGTEEL
LVKLNREDLLRKQRTFDNGSIPHQIHLGELHAILRRQEDFYPFLKDNREKIEKILTFRIPYYVGPLARGN
SRFAWMTRKSEETITPWNFEEVVDKGASAQSFIERMTNFDKNLPNEKVLPKHSLLYEYFTVYNELTKVKY
VTEGMRKPAFLSGEQKKAIVDLLFKTNRKVTVKQLKEDYFKKIECFDSVEISGVEDRFNASLGTYHDLLK
IIKDKDFLDNEENEDILEDIVLTLTLFEDREMIEERLKTYAHLFDDKVMKQLKRRRYTGWGRLSRKLING
IRDKQSGKTILDFLKSDGFANRNFMQLIHDDSLTFKEDIQKAQVSGQGDSLHEHIANLAGSPAIKKGILQ
TVKVVDELVKVMGRHKPENIVIEMARENQTTQKGQKNSRERMKRIEEGIKELGSQILKEHPVENTQLQNE
KLYLYYLQNGRDMYVDQELDINRLSDYDVDHIVPQSFLKDDSIDNKVLTRSDKNRGKSDNVPSEEVVKKM
KNYWRQLLNAKLITQRKFDNLTKAERGGLSELDKAGFIKRQLVETRQITKHVAQILDSRMNTKYDENDKL
IREVKVITLKSKLVSDFRKDFQFYKVREINNYHHAHDAYLNAVVGTALIKKYPKLESEFVYGDYKVYDVR
KMIAKSEQEIGKATAKYFFYSNIMNFFKTEITLANGEIRKRPLIETNGETGEIVWDKGRDFATVRKVLSM
PQVNIVKKTEVQTGGFSKESILPKRNSDKLIARKKDWDPKKYGGFDSPTVAYSVLVVAKVEKGKSKKLKS
VKELLGITIMERSSFEKNPIDFLEAKGYKEVKKDLIIKLPKYSLFELENGRKRMLASAGELQKGNELALP
SKYVNFLYLASHYEKLKGSPEDNEQKQLFVEQHKHYLDEIIEQISEFSKRVILADANLDKVLSAYNKHRD
KPIREQAENIIHLFTLTNLGAPAAFKYFDTTIDRKR**YTSTKEVLDATLIHQSITGLYETRIDLSQLGGD**G
TGG**PKKKRKVYPYDVPDYA**G**YPYDVPDYA**GS**YPYDVPDYA**GS**AAPAAKKKKLD**FESGM**PKKKRKV**AAFKP
NPINYILGLAIGIASVGWAMVEIDEDENPICLIDLGVRVFERAEVPKTGDSLAMARRLARSVRRLTRRRA
HRLLRARRLLKREGVLQAADFDENGLIKSLPNTPWQLRAAALDRKLTPLEWSAVLLHLIKHRGYLSQRKN
EGETADKELGALLKGVADNAHALQTGDFRTPAELALNKFEKESGHIRNQRGDYSHTFSRKDLQAELILLF
EKQKEFGNPHVSGGLKEGIETLLMTQRPALSGDAVQKMLGHCTFEPAEPKAAKNTYTAERFIWLTKLNNL
RILEQGSERPLTDTERATLMDEPYRKSKLTYAQARKLLGLEDTAFFKGLRYGKDNAEASTLMEMKAYHAI
SRALEKEGLKDKKSPLNLSPELQDEIGTAFSLFKTDEDITGRLKDRIQPEILEALLKHISFDKFVQISLK
ALRRIVPLMEQGKRYDEACAEIYGDHYGKKNTEEKIYLPPIPADEIRNPVVLRALSQARKVINGVVRRYG
SPARIHIETAREVGKSFKDRKEIEKRQEENRKDREKAAAKFREYFPNFVGEPKSKDILKLRLYEQQHGKC
LYSGKEINLGRLNEKGYVEIAAALPFSRTWDDSFNNKVLVLGSEAQNKGNQTPYEYFNGKDNSREWQEFK
ARVETSRFPRSKKQRILLQKFDEDGFKERNLNDTRYVNRFLCQFVADRMRLTGKGKKRVFASNGQITNLL
RGFWGLRKVRAENDRHHALDAVVVACSTVAMQQKITRFVRYKEMNAFDGKTIDKETGEVLHQKTHFPQPW
EFFAQEVMIRVFGKPDGKPEFEEADTPEKLRTLLAEKLSSRPEAVHEYVTPLFVSRAPNRKMSGQGHMET
VKSAKRLDEGVSVLRVPLTQLKLKDLEKMVNREREPKLYEALKARLEAHKDDPAKAFAEPFYKYDKAGNR
TQQVKAVRVEQVQKTGVWVRNHNGIADNATMVRVDVFEKGDKYYLVPIYSWQVAKGILPDRAVVQGKDEE
DWQLIDDSFNFKFSLHPNDLVEVITKKARMFGYFASCHRGTGNINIRIHDLDHKIGKNGILEGIGVKTAL
SFQKYQIDELGKEIRPCRLKKRPPVRSRAD**PKKKRKV**EASLE**KRPAATKKAGQAKKKK**GS

>SpCas9^{WT} -SaCas9^{WT}

M**KRPAATKKAGQAKKKK**LDGSGS**PKKKRKV**PW**DKKYSIGLDIGTNSVGWAVITDEYKVPSKKFKVLGNTD**
RHSIKKNLIGALLFDSGETAEATRLKRTARRRYTRRKNRICYLQEIFSNEMAKVDDSFFHRLEESFLVEE
DKKHERHPIFGNIVDEVAYHEKYPTIYHLRKKLVDSTDKADLRLIYLALAHMIKFRGHFLIEGDLNPDNS
DVDKLFIQLVQTYNQLFEENPINASGVDAKAILSARLSKSRRLENLIAQLPGEKKNGLFGNLIALSLGLT
PNFKSNFDLAEDAKLQLSKDTYDDDLDNLLAQIGDQYADLFLAAKNLSDAILLSDILRVNTEITKAPLSA
SMIKRYDEHHQDLTLLKALVRQQLPEKYKEIFFDQSKNGYAGYIDGGASQEEFYKFIKPILEKMDGTEEL
LVKLNREDLLRKQRTFDNGSIPHQIHLGELHAILRRQEDFYPFLKDNREKIEKILTFRIPYYVGPLARGN
SRFAWMTRKSEETITPWNFEEVVDKGASAQSFIERMTNFDKNLPNEKVLPKHSLLYEYFTVYNELTKVKY
VTEGMRKPAFLSGEQKKAIVDLLFKTNRKVTVKQLKEDYFKKIECFDSVEISGVEDRFNASLGTYHDLLK
IIKDKDFLDNEENEDILEDIVLTLTLFEDREMIEERLKTYAHLFDDKVMKQLKRRRYTGWGRLSRKLING
IRDKQSGKTILDFLKSDGFANRNFMQLIHDDSLTFKEDIQKAQVSGQGDSLHEHIANLAGSPAIKKGILQ
TVKVVDELVKVMGRHKPENIVIEMARENQTTQKGQKNSRERMKRIEEGIKELGSQILKEHPVENTQLQNE
KLYLYYLQNGRDMYVDQELDINRLSDYDVDHIVPQSFLKDDSIDNKVLTRSDKNRGKSDNVPSEEVVKKM
KNYWRQLLNAKLITQRKFDNLTKAERGGLSELDKAGFIKRQLVETRQITKHVAQILDSRMNTKYDENDKL
IREVKVITLKSKLVSDFRKDFQFYKVREINNYHHAHDAYLNAVVGTALIKKYPKLESEFVYGDYKVYDVR
KMIAKSEQEIGKATAKYFFYSNIMNFFKTEITLANGEIRKRPLIETNGETGEIVWDKGRDFATVRKVLSM
PQVNIVKKTEVQTGGFSKESILPKRNSDKLIARKKDWDPKKYGGFDSPTVAYSVLVVAKVEKGKSKKLKS
VKELLGITIMERSSFEKNPIDFLEAKGYKEVKKDLIIKLPKYSLFELENGRKRMLASAGELQKGNELALP
SKYVNFLYLASHYEKLKGSPEDNEQKQLFVEQHKHYLDEIIEQISEFSKRVILADANLDKVLSAYNKHRD
KPIREQAENIIHLFTLTNLGAPAAFKYFDTTIDRKRYTSTKEVLDATLIHQSITGLYETRIDLSQLGGDA
SGTGG**PKKKRKVYPYDVPDYA**G**YPYDVPDYA**GS**YPYDVPDYA**GS**AAPAAKKKKLD**FESGTSGIHGVPAAK
RNYILGLDIGITSVGYGIIDYETRDVIDAGVRLFKEANVENNEGRRSKRGARRLKRRRRHRIQRVKKLLF
DYNLLTDHSELSGINPYEARVKGLSQKLSEEEFSAALLHLAKRRGVHNVNEVEEDTGNELSTKEQISRNS
KALEEKYVAELQLERLKKDGEVRGSINRFKTSDYVKEAKQLLKVQKAYHQLDQSFIDTYIDLLETRRTYY
EGPGEGSPFGWKDIKEWYEMLMGHCTYFPEELRSVKYAYNADLYNALNDLNNLVITRDENEKLEYYEKFQ
IIENVFKQKKKPTLKQIAKEILVNEEDIKGYRVTSTGKPEFTNLKVYHDIKDITARKEIIENAELLDQIA
KILTIYQSSEDIQEELTNLNSELTQEEIEQISNLKGYTGTHNLSLKAINLILDELWHTNDNQIAIFNRLK
LVPKKVDLSQQKEIPTTLVDDFILSPVVKRSFIQSIKVINAIIKKYGLPNDIIIELAREKNSKDAQKMIN
EMQKRNRQTNERIEEIIRTTGKENAKYLIEKIKLHDMQEGKCLYSLEAIPLEDLLNNPFNYEVDHIIPRS
VSFDNSFNNKVLVKQEENSKKGNRTPFQYLSSSDSKISYETFKKHILNLAKGKGRISKTKKEYLLEERDI
NRFSVQKDFINRNLVDTRYATRGLMNLLRSYFRVNNLDVKVKSINGGFTSFLRRKWKFKKERNKGYKHHA
EDALIIANADFIFKEWKKLDKAKKVMENQMFEEKQAESMPEIETEQEYKEIFITPHQIKHIKDFKDYKYS
HRVDKKPNRELINDTLYSTRKDDKGNTLIVNNLNGLYDKDNDKLKKLINKSPEKLLMYHHDPQTYQKLKL
IMEQYGDEKNPLYKYYEETGNYLTKYSKKDNGPVIKKIKYYGNKLNAHLDITDDYPNSRNKVVKLSLKPY
RFDVYLDNGVYKFVTVKNLDVIKKENYYEVNSKCYEEAKKLKKISNQAEFIASFYNNDLIKINGELYRVI
GVNNDLLNRIEVNMIDITYREYLENMNDKRPPRIIKTIASKTQSIKKYSTDILGNLYEVKSKKHPQIIKK
GPG**KRPAATKKAGQAKKKK**LDGSGS**PAAKKKKLD**LE

>**SpCas9^{MT2}** -SaCas9^{WT}

M**KRPAATKKAGQAKKKK**LDGSGS**PKKKRKV**PW**DKKYSIGLDIGTNSVGWAVITDEYKVPSKKFKVLGNTD**
RHSIKKNLIGALLFDSGETAEATRLKRTARRRYTRRKNRICYLQEIFSNEMAKVDDSFFHRLEESFLVEE
DKKHERHPIFGNIVDEVAYHEKYPTIYHLRKKLVDSTDKADLRLIYLALAHMIKFRGHFLIEGDLNPDNS
DVDKLFIQLVQTYNQLFEENPINASGVDAKAILSARLSKSRRLENLIAQLPGEKKNGLFGNLIALSLGLT
PNFKSNFDLAEDAKLQLSKDTYDDDLDNLLAQIGDQYADLFLAAKNLSDAILLSDILRVNTEITKAPLSA
SMIKRYDEHHQDLTLLKALVRQQLPEKYKEIFFDQSKNGYAGYIDGGASQEEFYKFIKPILEKMDGTEEL
LVKLNREDLLRKQRTFDNGSIPHQIHLGELHAILRRQEDFYPFLKDNREKIEKILTFRIPYYVGPLARGN
SRFAWMTRKSEETITPWNFEEVVDKGASAQSFIERMTNFDKNLPNEKVLPKHSLLYEYFTVYNELTKVKY
VTEGMRKPAFLSGEQKKAIVDLLFKTNRKVTVKQLKEDYFKKIECFDSVEISGVEDRFNASLGTYHDLLK
IIKDKDFLDNEENEDILEDIVLTLTLFEDREMIEERLKTYAHLFDDKVMKQLKRRRYTGWGRLSRKLING
IRDKQSGKTILDFLKSDGFANRNFMQLIHDDSLTFKEDIQKAQVSGQGDSLHEHIANLAGSPAIKKGILQ
TVKVVDELVKVMGRHKPENIVIEMARENQTTQKGQKNSRERMKRIEEGIKELGSQILKEHPVENTQLQNE
KLYLYYLQNGRDMYVDQELDINRLSDYDVDHIVPQSFLKDDSIDNKVLTRSDKNRGKSDNVPSEEVVKKM
KNYWRQLLNAKLITQRKFDNLTKAERGGLSELDKAGFIKRQLVETRQITKHVAQILDSRMNTKYDENDKL
IREVKVITLKSKLVSDFRKDFQFYKVREINNYHHAHDAYLNAVVGTALIKKYPKLESEFVYGDYKVYDVR
KMIAKSEQEIGKATAKYFFYSNIMNFFKTEITLANGEIRKRPLIETNGETGEIVWDKGRDFATVRKVLSM
PQVNIVKKTEVQTGGFSKESILPKRNSDKLIARKKDWDPKKYGGFDSPTVAYSVLVVAKVEKGKSKKLKS
VKELLGITIMERSSFEKNPIDFLEAKGYKEVKKDLIIKLPKYSLFELENGRKRMLASAGELQKGNELALP
SKYVNFLYLASHYEKLKGSPEDNEQKQLFVEQHKHYLDEIIEQISEFSKRVILADANLDKVLSAYNKHRD
KPIREQAENIIHLFTLTNLGAPAAFKYFDTTIDSKRYTSTKEVLDATLIHQSITGLYETRIDLSQLGGDA
SGTGG**PKKKRKVYPYDVPDYA**G**YPYDVPDYA**GS**YPYDVPDYA**GS**AAPAAKKKKLD**FESGTSGIHGVPAAK
RNYILGLDIGITSVGYGIIDYETRDVIDAGVRLFKEANVENNEGRRSKRGARRLKRRRRHRIQRVKKLLF
DYNLLTDHSELSGINPYEARVKGLSQKLSEEEFSAALLHLAKRRGVHNVNEVEEDTGNELSTKEQISRNS
KALEEKYVAELQLERLKKDGEVRGSINRFKTSDYVKEAKQLLKVQKAYHQLDQSFIDTYIDLLETRRTYY
EGPGEGSPFGWKDIKEWYEMLMGHCTYFPEELRSVKYAYNADLYNALNDLNNLVITRDENEKLEYYEKFQ
IIENVFKQKKKPTLKQIAKEILVNEEDIKGYRVTSTGKPEFTNLKVYHDIKDITARKEIIENAELLDQIA
KILTIYQSSEDIQEELTNLNSELTQEEIEQISNLKGYTGTHNLSLKAINLILDELWHTNDNQIAIFNRLK
LVPKKVDLSQQKEIPTTLVDDFILSPVVKRSFIQSIKVINAIIKKYGLPNDIIIELAREKNSKDAQKMIN
EMQKRNRQTNERIEEIIRTTGKENAKYLIEKIKLHDMQEGKCLYSLEAIPLEDLLNNPFNYEVDHIIPRS
VSFDNSFNNKVLVKQEENSKKGNRTPFQYLSSSDSKISYETFKKHILNLAKGKGRISKTKKEYLLEERDI
NRFSVQKDFINRNLVDTRYATRGLMNLLRSYFRVNNLDVKVKSINGGFTSFLRRKWKFKKERNKGYKHHA
EDALIIANADFIFKEWKKLDKAKKVMENQMFEEKQAESMPEIETEQEYKEIFITPHQIKHIKDFKDYKYS
HRVDKKPNRELINDTLYSTRKDDKGNTLIVNNLNGLYDKDNDKLKKLINKSPEKLLMYHHDPQTYQKLKL
IMEQYGDEKNPLYKYYEETGNYLTKYSKKDNGPVIKKIKYYGNKLNAHLDITDDYPNSRNKVVKLSLKPY
RFDVYLDNGVYKFVTVKNLDVIKKENYYEVNSKCYEEAKKLKKISNQAEFIASFYNNDLIKINGELYRVI
GVNNDLLNRIEVNMIDITYREYLENMNDKRPPRIIKTIASKTQSIKKYSTDILGNLYEVKSKKHPQIIKK
GPG**KRPAATKKAGQAKKKK**LDGSGS**PAAKKKKLD**LE

>SpCas9^{MT3} -SaCas9^{WT}

MKRPAATKKAGQAKKKKLDGSGSPKKKRKVPWDKKYSIGLDIGTNSVGWAVITDEYKVPSKKFKVLGNTD
RHSIKKNLIGALLFDSGETAEATRLKRTARRRYTRRKNRICYLQEIFSNEMAKVDDSFFHRLEESFLVEE
DKKHERHPIFGNIVDEVAYHEKYPTIYHLRKKLVDSTDKADLRLIYLALAHMIKFRGHFLIEGDLNPDNS
DVDKLFIQLVQTYNQLFEENPINASGVDAKAILSARLSKSRRLENLIAQLPGEKKNGLFGNLIALSLGLT
PNFKSNFDLAEDAKLQLSKDTYDDDLDNLLAQIGDQYADLFLAAKNLSDAILLSDILRVNTEITKAPLSA
SMIKRYDEHHQDLTLLKALVRQQLPEKYKEIFFDQSKNGYAGYIDGGASQEEFYKFIKPILEKMDGTEEL
LVKLNREDLLRKQRTFDNGSIPHQIHLGELHAILRRQEDFYPFLKDNREKIEKILTFRIPYYVGPLARGN
SRFAWMTRKSEETITPWNFEEVVDKGASAQSFIERMTNFDKNLPNEKVLPKHSLLYEYFTVYNELTKVKY
VTEGMRKPAFLSGEQKKAIVDLLFKTNRKVTVKQLKEDYFKKIECFDSVEISGVEDRFNASLGTYHDLLK
IIKDKDFLDNEENEDILEDIVLTLTLFEDREMIEERLKTYAHLFDDKVMKQLKRRRYTGWGRLSRKLING
IRDKQSGKTILDFLKSDGFANRNFMQLIHDDSLTFKEDIQKAQVSGQGDSLHEHIANLAGSPAIKKGILQ
TVKVVDELVKVMGRHKPENIVIEMARENQTTQKGQKNSRERMKRIEEGIKELGSQILKEHPVENTQLQNE
KLYLYYLQNGRDMYVDQELDINRLSDYDVDHIVPQSFLKDDSIDNKVLTRSDKNRGKSDNVPSEEVVKKM
KNYWRQLLNAKLITQRKFDNLTKAERGGLSELDKAGFIKRQLVETRQITKHVAQILDSRMNTKYDENDKL
IREVKVITLKSKLVSDFRKDFQFYKVREINNYHHAHDAYLNAVVGTALIKKYPKLESEFVYGDYKVYDVR
KMIAKSEQEIGKATAKYFFYSNIMNFFKTEITLANGEIRKRPLIETNGETGEIVWDKGRDFATVRKVLSM
PQVNIVKKTEVQTGGFSKESILPKRNSDKLIARKKDWDPKKYGGFDSPTVAYSVLVVAKVEKGKSKKLKS
VKELLGITIMERSSFEKNPIDFLEAKGYKEVKKDLIIKLPKYSLFELENGRKRMLASAGELQKGNELALP
SKYVNFLYLASHYEKLKGSPEDNEQKQLFVEQHKHYLDEIIEQISEFSKRVILADANLDKVLSAYNKHRD
KPIREQAENIIHLFTLTNLGAPAAFKYFDTTIDRKKYTSTKEVLDATLIHQSITGLYETRIDLSQLGGDA
SGTGGPKKKRKVYPYDVPDYAGYPYDVPDYAGSYPYDVPDYAGSAAPAAKKKKLDFESGTSGIHGVPAAK
RNYILGLDIGITSVGYGIIDYETRDVIDAGVRLFKEANVENNEGRRSKRGARRLKRRRRHRIQRVKKLLF
DYNLLTDHSELSGINPYEARVKGLSQKLSEEEFSAALLHLAKRRGVHNVNEVEEDTGNELSTKEQISRNS
KALEEKYVAELQLERLKKDGEVRGSINRFKTSDYVKEAKQLLKVQKAYHQLDQSFIDTYIDLLETRRTYY
EGPGEGSPFGWKDIKEWYEMLMGHCTYFPEELRSVKYAYNADLYNALNDLNNLVITRDENEKLEYYEKFQ
IIENVFKQKKKPTLKQIAKEILVNEEDIKGYRVTSTGKPEFTNLKVYHDIKDITARKEIIENAELLDQIA
KILTIYQSSEDIQEELTNLNSELTQEEIEQISNLKGYTGTHNLSLKAINLILDELWHTNDNQIAIFNRLK
LVPKKVDLSQQKEIPTTLVDDFILSPVVKRSFIQSIKVINAIIKKYGLPNDIIIELAREKNSKDAQKMIN
EMQKRNRQTNERIEEIIRTTGKENAKYLIEKIKLHDMQEGKCLYSLEAIPLEDLLNNPFNYEVDHIIPRS
VSFDNSFNNKVLVKQEENSKKGNRTPFQYLSSSDSKISYETFKKHILNLAKGKGRISKTKKEYLLEERDI
NRFSVQKDFINRNLVDTRYATRGLMNLLRSYFRVNNLDVKVKSINGGFTSFLRRKWKFKKERNKGYKHHA
EDALIIANADFIFKEWKKLDKAKKVMENQMFEEKQAESMPEIETEQEYKEIFITPHQIKHIKDFKDYKYS
HRVDKKPNRELINDTLYSTRKDDKGNTLIVNNLNGLYDKDNDKLKKLINKSPEKLLMYHHDPQTYQKLKL
IMEQYGDEKNPLYKYYEETGNYLTKYSKKDNGPVIKKIKYYGNKLNAHLDITDDYPNSRNKVVKLSLKPY
RFDVYLDNGVYKFVTVKNLDVIKKENYYEVNSKCYEEAKKLKKISNQAEFIASFYNNDLIKINGELYRVI
GVNNDLLNRIEVNMIDITYREYLENMNDKRPPRIIKTIASKTQSIKKYSTDILGNLYEVKSKKHPQIIKK
GPGKRPAATKKAGQAKKKKLDGSGSPAAKKKKLDLE

>SpCas9^{WT} -dSaCas9

MKRPAATKKAGQAKKKKLDGSGSPKKKRKVPWDKKYSIGLDIGTNSVGWAVITDEYKVPSKKFKVLGNTD
RHSIKKNLIGALLFDSGETAEATRLKRTARRRYTRRKNRICYLQEIFSNEMAKVDDSFFHRLEESFLVEE
DKKHERHPIFGNIVDEVAYHEKYPTIYHLRKKLVDSTDKADLRLIYLALAHMIKFRGHFLIEGDLNPDNS
DVDKLFIQLVQTYNQLFEENPINASGVDAKAILSARLSKSRRLENLIAQLPGEKKNGLFGNLIALSLGLT
PNFKSNFDLAEDAKLQLSKDTYDDDLDNLLAQIGDQYADLFLAAKNLSDAILLSDILRVNTEITKAPLSA
SMIKRYDEHHQDLTLLKALVRQQLPEKYKEIFFDQSKNGYAGYIDGGASQEEFYKFIKPILEKMDGTEEL
LVKLNREDLLRKQRTFDNGSIPHQIHLGELHAILRRQEDFYPFLKDNREKIEKILTFRIPYYVGPLARGN
SRFAWMTRKSEETITPWNFEEVVDKGASAQSFIERMTNFDKNLPNEKVLPKHSLLYEYFTVYNELTKVKY
VTEGMRKPAFLSGEQKKAIVDLLFKTNRKVTVKQLKEDYFKKIECFDSVEISGVEDRFNASLGTYHDLLK
IIKDKDFLDNEENEDILEDIVLTLTLFEDREMIEERLKTYAHLFDDKVMKQLKRRRYTGWGRLSRKLING
IRDKQSGKTILDFLKSDGFANRNFMQLIHDDSLTFKEDIQKAQVSGQGDSLHEHIANLAGSPAIKKGILQ
TVKVVDELVKVMGRHKPENIVIEMARENQTTQKGQKNSRERMKRIEEGIKELGSQILKEHPVENTQLQNE
KLYLYYLQNGRDMYVDQELDINRLSDYDVDHIVPQSFLKDDSIDNKVLTRSDKNRGKSDNVPSEEVVKKM
KNYWRQLLNAKLITQRKFDNLTKAERGGLSELDKAGFIKRQLVETRQITKHVAQILDSRMNTKYDENDKL
IREVKVITLKSKLVSDFRKDFQFYKVREINNYHHAHDAYLNAVVGTALIKKYPKLESEFVYGDYKVYDVR
KMIAKSEQEIGKATAKYFFYSNIMNFFKTEITLANGEIRKRPLIETNGETGEIVWDKGRDFATVRKVLSM
PQVNIVKKTEVQTGGFSKESILPKRNSDKLIARKKDWDPKKYGGFDSPTVAYSVLVVAKVEKGKSKKLKS
VKELLGITIMERSSFEKNPIDFLEAKGYKEVKKDLIIKLPKYSLFELENGRKRMLASAGELQKGNELALP
SKYVNFLYLASHYEKLKGSPEDNEQKQLFVEQHKHYLDEIIEQISEFSKRVILADANLDKVLSAYNKHRD
KPIREQAENIIHLFTLTNLGAPAAFKYFDTTIDRKRYTSTKEVLDATLIHQSITGLYETRIDLSQLGGDA
SGTGGPKKKRKVYPYDVPDYAGYPYDVPDYAGSYPYDVPDYAGSAAPAAKKKKLDFESGTSGIHGVPAAK
RNYILGLAIGITSVGYGIIDYETRDVIDAGVRLFKEANVENNEGRRSKRGARRLKRRRRHRIQRVKKLLF
DYNLLTDHSELSGINPYEARVKGLSQKLSEEEFSAALLHLAKRRGVHNVNEVEEDTGNELSTKEQISRNS
KALEEKYVAELQLERLKKDGEVRGSINRFKTSDYVKEAKQLLKVQKAYHQLDQSFIDTYIDLLETRRTYY
EGPGEGSPFGWKDIKEWYEMLMGHCTYFPEELRSVKYAYNADLYNALNDLNNLVITRDENEKLEYYEKFQ
IIENVFKQKKKPTLKQIAKEILVNEEDIKGYRVTSTGKPEFTNLKVYHDIKDITARKEIIENAELLDQIA
KILTIYQSSEDIQEELTNLNSELTQEEIEQISNLKGYTGTHNLSLKAINLILDELWHTNDNQIAIFNRLK
LVPKKVDLSQQKEIPTTLVDDFILSPVVKRSFIQSIKVINAIIKKYGLPNDIIIELAREKNSKDAQKMIN
EMQKRNRQTNERIEEIIRTTGKENAKYLIEKIKLHDMQEGKCLYSLEAIPLEDLLNNPFNYEVDHIIPRS
VSFDNSFNNKVLVKQEEASKKGNRTPFQYLSSSDSKISYETFKKHILNLAKGKGRISKTKKEYLLEERDI
NRFSVQKDFINRNLVDTRYATRGLMNLLRSYFRVNNLDVKVKSINGGFTSFLRRKWKFKKERNKGYKHHA
EDALIIANADFIFKEWKKLDKAKKVMENQMFEEKQAESMPEIETEQEYKEIFITPHQIKHIKDFKDYKYS
HRVDKKPNRELINDTLYSTRKDDKGNTLIVNNLNGLYDKDNDKLKKLINKSPEKLLMYHHDPQTYQKLKL
IMEQYGDEKNPLYKYYEETGNYLTKYSKKDNGPVIKKIKYYGNKLNAHLDITDDYPNSRNKVVKLSLKPY
RFDVYLDNGVYKFVTVKNLDVIKKENYYEVNSKCYEEAKKLKKISNQAEFIASFYNNDLIKINGELYRVI
GVNNDLLNRIEVNMIDITYREYLENMNDKRPPRIIKTIASKTQSIKKYSTDILGNLYEVKSKKHPQIIKK
GPGKRPAATKKAGQAKKKKLDGSGSPAAKKKKLDLE

>**SpCas9**MT3 -dSaCas9

M**KRPAATKKAGQAKKKK**LDGSGS**PKKKRKV**PW**DKKYSIGLDIGTNSVGWAVITDEYKVPSKKFKVLGNTD**
RHSIKKNLIGALLFDSGETAEATRLKRTARRRYTRRKNRICYLQEIFSNEMAKVDDSFFHRLEESFLVEE
DKKHERHPIFGNIVDEVAYHEKYPTIYHLRKKLVDSTDKADLRLIYLALAHMIKFRGHFLIEGDLNPDNS
DVDKLFIQLVQTYNQLFEENPINASGVDAKAILSARLSKSRRLENLIAQLPGEKKNGLFGNLIALSLGLT
PNFKSNFDLAEDAKLQLSKDTYDDDLDNLLAQIGDQYADLFLAAKNLSDAILLSDILRVNTEITKAPLSA
SMIKRYDEHHQDLTLLKALVRQQLPEKYKEIFFDQSKNGYAGYIDGGASQEEFYKFIKPILEKMDGTEEL
LVKLNREDLLRKQRTFDNGSIPHQIHLGELHAILRRQEDFYPFLKDNREKIEKILTFRIPYYVGPLARGN
SRFAWMTRKSEETITPWNFEEVVDKGASAQSFIERMTNFDKNLPNEKVLPKHSLLYEYFTVYNELTKVKY
VTEGMRKPAFLSGEQKKAIVDLLFKTNRKVTVKQLKEDYFKKIECFDSVEISGVEDRFNASLGTYHDLLK
IIKDKDFLDNEENEDILEDIVLTLTLFEDREMIEERLKTYAHLFDDKVMKQLKRRRYTGWGRLSRKLING
IRDKQSGKTILDFLKSDGFANRNFMQLIHDDSLTFKEDIQKAQVSGQGDSLHEHIANLAGSPAIKKGILQ
TVKVVDELVKVMGRHKPENIVIEMARENQTTQKGQKNSRERMKRIEEGIKELGSQILKEHPVENTQLQNE
KLYLYYLQNGRDMYVDQELDINRLSDYDVDHIVPQSFLKDDSIDNKVLTRSDKNRGKSDNVPSEEVVKKM
KNYWRQLLNAKLITQRKFDNLTKAERGGLSELDKAGFIKRQLVETRQITKHVAQILDSRMNTKYDENDKL
IREVKVITLKSKLVSDFRKDFQFYKVREINNYHHAHDAYLNAVVGTALIKKYPKLESEFVYGDYKVYDVR
KMIAKSEQEIGKATAKYFFYSNIMNFFKTEITLANGEIRKRPLIETNGETGEIVWDKGRDFATVRKVLSM
PQVNIVKKTEVQTGGFSKESILPKRNSDKLIARKKDWDPKKYGGFDSPTVAYSVLVVAKVEKGKSKKLKS
VKELLGITIMERSSFEKNPIDFLEAKGYKEVKKDLIIKLPKYSLFELENGRKRMLASAGELQKGNELALP
SKYVNFLYLASHYEKLKGSPEDNEQKQLFVEQHKHYLDEIIEQISEFSKRVILADANLDKVLSAYNKHRD
KPIREQAENIIHLFTLTNLGAPAAFKYFDTTIDRKKYTSTKEVLDATLIHQSITGLYETRIDLSQLGGDA
SGTGG**PKKKRKV****YPYDVPDYA**G**YPYDVPDYA**GS**YPYDVPDYA**GS**AAPAAKKKKLD**FESGTSGIHGVPAAK
RNYILGLAIGITSVGYGIIDYETRDVIDAGVRLFKEANVENNEGRRSKRGARRLKRRRRHRIQRVKKLLF
DYNLLTDHSELSGINPYEARVKGLSQKLSEEEFSAALLHLAKRRGVHNVNEVEEDTGNELSTKEQISRNS
KALEEKYVAELQLERLKKDGEVRGSINRFKTSDYVKEAKQLLKVQKAYHQLDQSFIDTYIDLLETRRTYY
EGPGEGSPFGWKDIKEWYEMLMGHCTYFPEELRSVKYAYNADLYNALNDLNNLVITRDENEKLEYYEKFQ
IIENVFKQKKKPTLKQIAKEILVNEEDIKGYRVTSTGKPEFTNLKVYHDIKDITARKEIIENAELLDQIA
KILTIYQSSEDIQEELTNLNSELTQEEIEQISNLKGYTGTHNLSLKAINLILDELWHTNDNQIAIFNRLK
LVPKKVDLSQQKEIPTTLVDDFILSPVVKRSFIQSIKVINAIIKKYGLPNDIIIELAREKNSKDAQKMIN
EMQKRNRQTNERIEEIIRTTGKENAKYLIEKIKLHDMQEGKCLYSLEAIPLEDLLNNPFNYEVDHIIPRS
VSFDNSFNNKVLVKQEEASKKGNRTPFQYLSSSDSKISYETFKKHILNLAKGKGRISKTKKEYLLEERDI
NRFSVQKDFINRNLVDTRYATRGLMNLLRSYFRVNNLDVKVKSINGGFTSFLRRKWKFKKERNKGYKHHA
EDALIIANADFIFKEWKKLDKAKKVMENQMFEEKQAESMPEIETEQEYKEIFITPHQIKHIKDFKDYKYS
HRVDKKPNRELINDTLYSTRKDDKGNTLIVNNLNGLYDKDNDKLKKLINKSPEKLLMYHHDPQTYQKLKL
IMEQYGDEKNPLYKYYEETGNYLTKYSKKDNGPVIKKIKYYGNKLNAHLDITDDYPNSRNKVVKLSLKPY
RFDVYLDNGVYKFVTVKNLDVIKKENYYEVNSKCYEEAKKLKKISNQAEFIASFYNNDLIKINGELYRVI
GVNNDLLNRIEVNMIDITYREYLENMNDKRPPRIIKTIASKTQSIKKYSTDILGNLYEVKSKKHPQIIKK
GPG**KRPAATKKAGQAKKKK**LDGSGS**PAAKKKKLD**LE
